# Novel Sources of Tolerance to Aluminium Toxicity in Wild *Cicer* (*Cicer reticulatum* and *Cicer echinospermum*) Collections

**DOI:** 10.3389/fpls.2021.678211

**Published:** 2021-06-25

**Authors:** Wendy Vance, Karthika Pradeep, Scott R. Strachan, Simon Diffey, Richard W. Bell

**Affiliations:** ^1^Centre for Sustainable Farming Systems, Future Food Institute, Murdoch University, Perth, WA, Australia; ^2^Apex Biometry, South Fremantle, WA, Australia

**Keywords:** chickpea, solution culture, aluminium tolerance, wild *Cicer*, genotypic variation, genetic population groups

## Abstract

In acid soils, the toxic form of aluminium, Al^3+^, significantly inhibits root growth and elongation, leading to less water and nutrient uptake. Previous research had shown differential Al toxicity tolerance among cultivated *Cicer arietinum* L. (chickpea); however, the potential for developing tolerant cultivars is limited by the narrow genetic diversity of cultivated chickpeas. Recent collections from Turkey of wild *Cicer* species, *Cicer reticulatum*, and *Cicer echinospermum*, have increased the available gene pool significantly, but there has been no large-scale screening of wild *Cicer* for acid tolerance or Al^3+^ toxicity tolerance. This study evaluated 167 wild *Cicer* and 17 Australian chickpea cultivars in a series of screenings under controlled growth conditions. The pH of 4.2 and Al concentrations of 15 and 60 μM Al were selected for large-scale screening based on dose response experiments in a low ionic strength nutrient solution. The change in root length showed better discrimination between tolerant and sensitive lines when compared with shoot and root dry weights and was used as a selection criterion. In a large-scale screening, 13 wild *Cicer reticulatum* accessions had a higher root tolerance index (≥50%), and eight had higher relative change in root length (≥40%) compared with PBA Monarch, which showed greater tolerance among the Australian domestic cultivars screened. In general, *C. reticulatum* species were found to be more tolerant than *C. echinospermum*, while genetic population groups Ret_5, Ret_6, and Ret_7 from Diyarbakir and Mardin Province were more tolerant than other groups. Among *C. echinospermum*, Ech_6 from the Siv-Diyar collection site of the Urfa Province showed better tolerance than other groups. In this first detailed screening of aluminium toxicity tolerance in the new wild *Cicer* collections, we identified accessions that were more tolerant than current domestic cultivars, providing promising germplasm for breeding programs to expand chickpea adaptation to acid soils.

## Introduction

Chickpea (*Cicer arietinum* L.) is the largest pulse crop after common bean, with annual global production of 17.2 million tonnes from 17.8 Mha, and is a primary source of food protein for about 20% of the world population (Vadez et al., 2020). India is the largest chickpea producer, accounting for about 65%, followed by Australia (14%), Myanmar (4%), Ethiopia (4%), Turkey (3%), Russia (3%), Iran, USA, Pakistan (2% each), and other important countries include Mexico, Morocco, and Syria (Merga and Haji, 2019). Due to the demands of an increasing population and its nutritional value, the outlook for chickpea expansion is excellent, but, presently, it is only recommended for soils with a pH of 6–9 (Ahlawat et al., 2007). In India, 30% of cultivated land is considered acidic (Kumar et al., 2014) and, in Australia, surface and subsoil acidity compromise 50% of agricultural land (Moroni et al., 2010). The widespread occurrence of soil acidity is one of the main limitations to chickpea production worldwide. On most of the acid soils, toxic levels of aluminium (Al), manganese (Mn), along with P deficiency are the limiting factors for plant growth (Kochian et al., 2005). Lime application is commonly used to manage soil acidity, while the addition of P-containing fertilisers can increase bioavailable P and reduce Al^3+^ toxicity (Liao et al., 2006). These methods are not always economically feasible, and lime application is ineffective in acid subsoil without deep tillage (Dai et al., 2011). An effective approach is to develop acid tolerant cultivars to increase crop productivity. Despite being an important pulse crop, no acid tolerant chickpea cultivars have been developed. In order to breed cultivars for acid tolerance, the first step is to identify Al-tolerant genetic resources (Foy et al., 1967).

Domesticated chickpeas are very limited in their genetic diversity, and, therefore, wild cultivars are important as a source of resistance to stressors (Berger, 2006). There is a potential to exploit crop wild relatives (CWR) in the *Cicer* genus and improve crop production. Crop wild relatives are closely related to domestic cultivars and include crop progenitors, landraces, and closely related taxa not involved in agriculture, which hold key sources of new genetic traits to develop improved crop lines through breeding (Ananda et al., 2020). There are nine annual *Cicer* species, and, among them, *Cicer reticulatum* and *Cicer echinospermum* are closely related to cultivated *Cicer arietinum* L. based on karyotype symmetry indices, and they are considered as wild progenitors due to successful previous crosses with *C. arietinum* L. (Singh and Ocampo, 1997). Wild *Cicer* species had better resistance to Ascochyta blight, (*Ascochytarabiei*) (Collard et al., 2003), fusarium wilt (*Fusarium oxysporum*), leaf miner (*Liriomyzacicerina*), seed beetle (*Callosobruchuschinensis* L.), cyst nematode (*Heteroderaciceri*), cold (Singh and Ocampo, 1997), and root lesion nematode (*Pratylenchusthornei*) (Reen et al., 2019). Also, better Mn toxicity tolerance in a *C. echinospermum* accession when compared with *C. reticulatum* has recently been reported (Pradeep et al., 2020). Other research on nutrient uptake between two wild species, *C. reticulatum* and *C. echinospermum*, showed that there was variability in an Al tolerance index and nutrient uptake among the cultivated and wild *Cicer* species (Sultana et al., 2020). However, there has been no large-scale exploitation of wild *Cicer* species for Al toxicity tolerance. Collections of wild *Cicer* germplasm may contain lines with Al tolerance (Berger, 2006). There is also limited research reported on tolerance of domestic chickpea cultivars to low pH and high-soluble Al conditions. The first step in developing chickpeas resistant to Al is to screen germplasm under Al-toxic conditions.

Previous studies on *C. arietinum* L. showed that Al^3+^ stress-inhibited germination, root growth, and biomass through oxidative stress and peroxidation of membrane lipids and loss of plasma membrane integrity (Singh and Raje, 2011; Choudhury and Sharma, 2014). The variability within chickpea to Al tolerance was attributed to Al exclusion, as there was reduced accumulation of Al in root apices (Singh and Raje, 2011; Ryan, 2018). Another study suggested that chickpea genotypes with efficient nitrate accumulation were more tolerant of Al stress (Sharma et al., 2015). However, the major drawback of these studies was the high solution ion concentrations. Nutrient solutions used to study Al toxicity should be ≤4.5 pH and have ≤5 μM phosphorus and low total ion concentration in order to accurately assess the toxic effects of Al for plant growth (Kopittke and Blamey, 2016). When appropriate solution concentrations are not used during solution screening, the levels of Al reported are inflated, as they do not account for speciation of Al into non-toxic forms, or complexing of Al to non-toxic forms with nutrients in the solution such as sulphate or phosphate.

The annual species, *C. reticulatum* and *C. echinospermum*, are the only CWR cross-compatible with domestic *Cicer* (Croser et al., 2003), and their occurrence is restricted to southeastern Anatolia in Turkey within orchards, vineyards, rocky slopes, forests, and fields. Moreover, there is a threat to the survival of these species due to climate change, urbanisation, and industrial developments (Talip et al., 2018; von Wettberg et al., 2018). Between 2013 and 2015, a new collection mission in Southeastern Turkey increased the number of the *C. reticulatum* and *C. echinospermum* accessions manifold (von Wettberg et al., 2018). Multiple studies showed that CWR of *Cicer* possess a superior tolerance to various parameters, making them a valuable resource for chickpea improvement (Abbo et al., 2003; Reen et al., 2019), and this paper explores the Al tolerance at low pH in CWR of *Cicer* and compares them with cultivars of *C. arietinum* L. The objectives of this study were to (i) determine the effect of low pH and Al on plant growth of Australian domesticated *Cicer* cultivars; (ii) characterise the response of wild *Cicer* accessions to growing at low solution pH with increasing Al to identify the range of tolerance; and (iii) determine if there is any differentiation in tolerance to low pH and Al among the *Cicer* accessions based on the species or genetic population group.

## Materials and Methods

### *Cicer* Accessions

There were 17 Australian chickpea cultivars (*C. arietinum* L.), with a range of yield potential; 14 desi type (Ambar, Genesis 079, Genesis 836, Kyabra, Maiden, Moti, Neelum, PBA Boundary, PBA HatTrick, PBA Pistol, PBA Seamer, PBA Slasher, PBA Striker, and Yorker); and 3 Kabuli type (Genesis 090, Kalkee, and PBA Monarch) and 167 wild *Cicer* accessions used in the experiments. Wild *Cicer* accessions were obtained from the Australian Grains Genebank. The species, the collection site in Turkey, the genetic population group (von Wettberg et al., 2018), and the prefix and suffix code numbers used to identify each accession are presented in Table 1. Of the 167 wild *Cicer* accessions screened, 127 were *C. reticulatum* (*C. retic*), and 40 were *C. echinospermum* (*C. echino*).

**Table 1:** List of wild *Cicer* accessions, their collection province and site, species, number of accessions from each site, and the prefix and suffix code numbers used to identify the population.

**Province, collection site**	**Species**	**Genetic population**	**No. of accessions**	**Prefix**	**Suffix and accession code number**
**Adiyaman**					
Oyali	*C. retic*	Ret_1	6	Oyali	071, 073, 076^*^, 084^*^, 100, 107^*^
**Diyarbakir**					
Kesentas	*C. retic*	Ret_2	10	Kesen	062, 065, 066^*^, 067, 071, 073, 075^*^, 077, 101, 104
Egil	*C. retic*	Ret_5	6	Egil	063, 065, 066^*^, 073^*^,074^*^, 075^*^
Kalkan	*C. retic*	Ret_5	6	Kalka	061, 064, 066, 067, 070, 074^*^
Gunasan	*C. echino*	Ech_6	2	Gunas	062, 100
Cermik	*C. echino*	Ech_7	6	Cermi	061^*^, 063^*^, 071^*^, 072^*^, 073, 075
**Mardin**					
Baristepe1	*C. retic*	Ret_8	8	Bari1	062, 063, 064, 068, 069, 091, 092, 093
Baristepe2	*C. retic*	Ret_7	5	Bari2	062, 064, 067, 072, 074
Baristepe3	*C. retic*	Ret_7	17	Bari3	064, 065, 067, 072C, 073, 074, 075, 079, 091, 092, 100, 101, 102, 103, 106D, 110, 112
Beslever	*C. retic*	Ret_6	8	Besev	061^*^, 062, 065, 066, 074, 075, 079, 083
Dereici	*C. retic*	Ret_6	10	Derei	062, 065, 066^*^, 069, 070, 072, 073, 074, 075, 078^*^
Kayatepe	*C. retic*	Ret_6	7	Kayat	061, 063, 064^*^, 066, 070, 077^*^, 080
Sarikaya	*C. retic*	Ret_6	10	Sarik	061^*^, 064^*^, 065^*^, 066^*^, 067, 073^*^, 074^*^, 077, 078, 080^*^
Savur	*C. retic*	Ret_6	1	Savur	063
**Sirnak**					
CudiB	*C. retic*	Ret_11	11	CudiB	004, 005, 006, 008B, 011, 016, 017^*^, 018^*^, 019, 022C, 023
CudiA	*C. retic*	Ret_11	14	CudiA	101A^*^, 103C, 104, 105, 122, 124, 127, 128, 151,152, 153, 154, 155, 221
Sirnak	*C. retic*	Ret_12	8	Sirna	060, 061^*^, 064^*^, 071C^*^, 083^*^, 084^*^, 085^*^, 104^*^
**Urfa**					
Destek	*C. echino*	Ech_5	9	Deste	061, 063, 064^*^, 066^*^, 071^*^, 072^*^, 073^*^, 075^*^, 080
Siv-Diyar	*C. echino*	Ech_6	9	S2Drd	061, 062, 065, 100, 101, 102, 104, 105, 107B
Karabahce	*C. echino*	Ech_8	12	Karab	062A, 063, 081^*^, 082^*^, 085^*^, 086, 091B^*^ 092, 162, 164^*^, 172, 174^*^
Ortanca	*C. echino*	Ech_9	2	Ortan	061, 066^*^

* *Accessions used in Experiment 4; C. retic, C. reticulatum; C. echino, C. echinospermum*.

### Growth Conditions and an Experimental Setup

All experiments were completed in solution culture in a growth cabinet with the temperature set to 22°C, and with 12-h light and dark periods. Seeds were sterilised in a 3% solution of sodium hypochlorite for 5 min before being rinsed five times with deionised water. Seeds were then germinated in the dark for 2 or 4 days at 22°C on a paper towel wet with tap water before being randomly allocated to a hydroponic solution. Lupin (*Lupinus angustifolius*) and cowpea (*Vigna unguiculata*) were used as low pH, Al-tolerant checks in the experiments (Choudhury and Sharma, 2014; Blamey et al., 2017). The experiments were arranged in a split-plot design with pH ± Al treatments as main plots and genotypes as subplots with three replicates. There were either five seeds (Experiment 1), 10 seeds (Experiment 2), or three seeds (Experiments 3–6) per genotype in each replicate unit for experiments. The plants were harvested 14 days after treatments in experiments 1 and 3, and 10 days after treatments in experiments 2, 4, 5, and 6. The treatments are detailed in Table 2. In experiments 4, 5, and 6, replicates were grown as consecutive runs due to limited space availability in the growth cabinet.

**Table 2:** Experiment description, genotypes, and pH and Al concentrations used in experiments.

**Experiment**	**Genotypes**	**pH treatments**	**Al levels**
Experiment 1—Dose response to pH	Ambar, Genesis 836, PBA HatTrick, PBA Slasher, and PBA Striker	6.5, 4.2, 3.8, 3.4, 3.2, and 3	Nil
Experiment 2—Dose response to pH and aluminium	Ambar, Genesis 836, PBA HatTrick, PBA Slasher, and PBA Striker	6.5 (only without Al) and 4.2 (0 to 90 μM Al)	0, 15, 30, 45, 60, 75, and 90 μM
Experiment 3—Dose response to pH of 4.2 and aluminium	PBA Monarch, Genesis 090, Kalkee, Moti, PBA Seamer, PBA Boundary, Kyabra, PBA Pistol, Yorker	4.2	0, 15, 30, and 60 μM
Experiment 4—Large scale screening of genotypes	49 wild *Cicer*, and 17 domestic cultivars; Ambar, PBA Striker; lupin and cowpea as checks	4.2	0, 15, and 60 μM
Experiment 5—Large scale screening of genotypes	118 wild *Cicer* accessions Ambar, PBA Striker, and lupin as checks	4.2	0, 15, and 60 μM
Experiment 6—Confirmation screening	A selection of tolerant and sensitive genotypes from Experiment 4 and 5 42 wild *Cicer*, and 6 domestic cultivars: Ambar, PBA Striker, PBA HatTrick, PBA Seamer, Moti, PBA Slasher Lupin and cowpea as checks	4.2	0, 15, and 60 μM

### Experiments

Initial hydroponics experiments were completed to determine the limiting pH (Experiment 1) and the growth response to low pH with increasing Al concentrations (Experiments 2 and 3) with *C. arietinum* L. cultivars. From these results, the Al concentrations to be used in the larger screening experiments in a solution of pH 4.2 for the wild *Cicer* were determined. Experiments 4–6 determined the response of wild *Cicer* accessions to concentrations of Al at pH 4.2 in solution culture; experiments 4 and 5 were large-scale screens conducted at different times, with the inclusion of Ambar, PBA Striker, and lupin as experiment checks in common. Experiment 6 was a confirmation screen with a selection of tolerant and sensitive accessions from experiments 4 and 5. Table 2 details the genotypes, pH, and Al concentrations used in each experiment.

### Solution Composition

The hydroponic solution contained macronutrients, micronutrients, phosphorus, iron, and EDTA (μM): KNO_3_, 650; CaCl_2_.2H_2_O, 400; MgCl_2_.6H_2_O, 250; NH_4_NO_3_, 40; H_3_BO_3_, 23; (NH_4_)_2_SO_4_, 10; MnCl_2_.4H_2_O, 9; ZnSO_4_.7H_2_O, 0.8; CuSO_4_.5H_2_O, 0.3; Na_2_MoO_4_.2H_2_O, 0.1; Na_2_HPO_4_, 13 (Experiments 1–3) or 5 (Experiments 4 and 5). Iron (20 μM) was supplied as Fe-EDTA prepared from equimolar amounts of FeCl_3_.6H_2_O, and Na_2_EDTA. Composition of solution was based on recommendations from the literature (Blamey et al., 1991; Hede et al., 2001; Famoso et al., 2010; Moroni et al., 2010; Kopittke and Blamey, 2016). In experiments where Al was a treatment, it was added as AlCl_3_.6H_2_O. The solution was changed every 2 days throughout the experiment (solution was sampled before and after use).

### Measurements

The plant parameters measured were longest root length (LLR) (except in Experiment 2), shoot and root dry weight (60°C for 48 h). The methods followed were the length of root post-germination and length of the longest root at harvest (mm) (LLR) measured by a ruler (mm increments) or Vernier calliper. Relative shoot growth (RSG) and relative root growth (RRG) were calculated from the dry weight treated/dry weight mean control. Indices of root tolerance were calculated as root tolerance index (RTI) (LLR treated/LLR mean control) and relative change in root length (RRL) (net root length mean treated/net root length mean control).

The nutrient solutions were collected every 2 days when the solution was changed (both solutions that went into and came out of treatment containers). The 50 ml samples were tested for pH before 10 ml was filtered (0.45 μm) and acidified for analysis of Al by inductively coupled plasma atomic emission spectroscopy (ICP-IES). The ionic concentration, activity of Al^3+^, and the expected free Al^3+^ concentration of the solutions were calculated, using the chemical speciation program Geochem-EZ (Shaff et al., 2010).

### Statistical Analysis

Experiments 1, 2, and 3: analysis of variance was used (ANOVA). The least significant difference (LSD) at the 5% level was used to show differences among means. All analysis was carried out with GenStat v18 (VSN International Ltd, United Kingdom). Plots of residuals were used to check that the assumptions underlying analysis of variance were observed (residuals are normally and independently distributed with common variance). When necessary, the data were transformed so that these assumptions were met. In the paper, means presented are the back transformed adjusted values. When shoot and root weight required a log transformation, the following was used: log shoot weight = log (shoot weight +0.1); log root weight = log (root weight +0.1). Note that the added constant was chosen independently for each measurement as required. Regression analysis was also used to determine if there were differences among cultivars in the growth response.

Experiments 4 and 6: The data were analysed, using linear mixed models. Linear mixed models can be formulated in such a way that they are analogous to ANOVA. However, linear mixed models have the added flexibility of accommodating missing values in the response and explanatory variables and can accommodate a wide range of variance models; the latter was important in this case where there was evidence of variance heterogeneity between containers and spatial variation within containers. The linear mixed model for each measurement was developed in two stages. In the first stage, the following baseline linear mixed model was fitted, using notation described by Wilkinson and Rogers (1973):

Trait ~ 1 + *Aluminium Treatment* + *Id* + *Aluminium Treatment.Id* + *Run* + *Run.Container* + *Run.Container.ContCol* + *Run.Container.ContRow* + *Run.Container.ContCol.ContRow* (M1)

where 1 is the overall mean and terms in the italic font are fitted as random effects. The term *Id* refers to individual accessions and *ContCol* and *ContRow* to a container column and a row, respectively. Associated with each random term is a variance parameter (often referred to as “a variance component”). The preferred method for estimating these parameters is residual (or restricted) maximum likelihood (REML) (Patterson and Thompson, 1971). All models were fitted, using the statistical software package ASReml within the R (R Core Team, 2018) computing environment. The term *Run.Container.ContCol.ContRow* indexes experimental units and is associated with the residual variance. This term is not explicitly fitted in the call to ASReml. In the second stage of model development, variance heterogeneity between containers and spatial variability within containers was considered. Spatial variability within containers was considered, using the first-order separable autoregressive models described by Cullis and Gleeson (1991).

Experiments 4 and 6 data were analysed based on accessions. Experiment 5 had 118 wild *Cicer* accessions analysed individually and also based on their genetic population groups. In the case of experiment 5, the genetic population information associated with each accession (see Table 1) was included in the model presented in (M1) by partitioning the term *Id* into *GenPop/Id* where/is the nesting operator and *GenPop/Id*=*GenPop*+*GenPop.Id*. The main effect of *GenPop* was fitted as a fixed effect as was the interaction of *GenPop* and *Al treatment*. The terms *GenPop.Id* and *Al Treatment.GenPop.Id* were fitted as random effects.

## Results

### Nutrient Solution Composition

The ionic strength of the solutions calculated by Geochem-EZ for Experiment 2 was between 2,820 and 3,290 μM, increasing with Al added to the solution. The initial solutions (and replacement solutions every 2 days) had Al concentrations close to the nominated Al concentration (Table 3). After 2 days, the proportion of Al decreased to 42–83% of the nominated 15–90 μM Al concentrations. Geochem-EZ analysis of the solution for the expected free Al concentration of each treatment was also close to the nominated Al concentration; the remainder of the Al was complexed with other compounds. Geochem-EZ was used to analyse the difference in expected free Al concentration between solutions with 13 μM PO_4_ (Experiments 1 to 3) and 5 μM PO_4_ (Experiments 4 and 5), the latter being the recommended concentration by Kopittke and Blamey (2016). The lower concentration of PO_4_ added to the solution decreased the proportion of Al complexed with PO_4_, leaving additional free Al in solution, and, hence, 5 μM of PO_4_ was used in all the following experiments.

**Table 3:** Comparison of the treatment Al and actual soluble Al and free Al concentration as modelled by Geochem-EZ (for Experiment 2), treatments at pH 4.2.

**Treatment concentration (μM)**	**Soluble Al in start solution (μM) (ICP-IES)**	**Soluble Al at 2 days (μM) (ICP-IES)**	**Geochem-EZ predicted Solution Al (μM)**	**Geochem-EZ predicted Al^**3+**^ Activity (μM)**	**Geochem-EZ predicted Ionic strength of solution (μM)**
0	–	–	–	–	2,820
15	14.4	6.0	11.0	6	2,890
30	28.0	17.6	22.6	13	2,970
45	43.7	30.0	34.5	20	3,050
60	57.1	42.6	46.6	26	3,130
75	73.4	57.7	58.9	33	3,210
90	88.9	74.6	71.4	40	3,290

The solutions used in other studies for chickpeas (Singh and Raje, 2011; Choudhury and Sharma, 2014), cereals (Hede et al., 2001, 2002) and barley (Moroni et al., 2010) were analysed by Geochem-EZ to predict Al concentration and ionic strength (data presented as Supplementary Figure A, Supplementary Table A). The solution for this screening was initially based on solutions by Moroni et al. (2010) (for micronutrients) and Hede et al. (2001); however, the solution by Moroni et al. (2010) had a high concentration of some macronutrients, high ionic strength, and higher P concentration than recommended by Kopittke and Blamey (2016), whereas, Hede et al. (2001) had lower concentrations of the macronutrients and a lower ionic strength. Among the two previous studies on chickpeas, Choudhury and Sharma (2014) had a simple, low ionic strength solution, and Singh and Raje (2011) had a high ionic strength, but, in the high Al concentrations in the latter solution, it is estimated by Geochem-EZ that 15% of the Al would not be available as Al^3+^.

### Symptoms of Low pH and Al Toxicity

**Figure 1:**
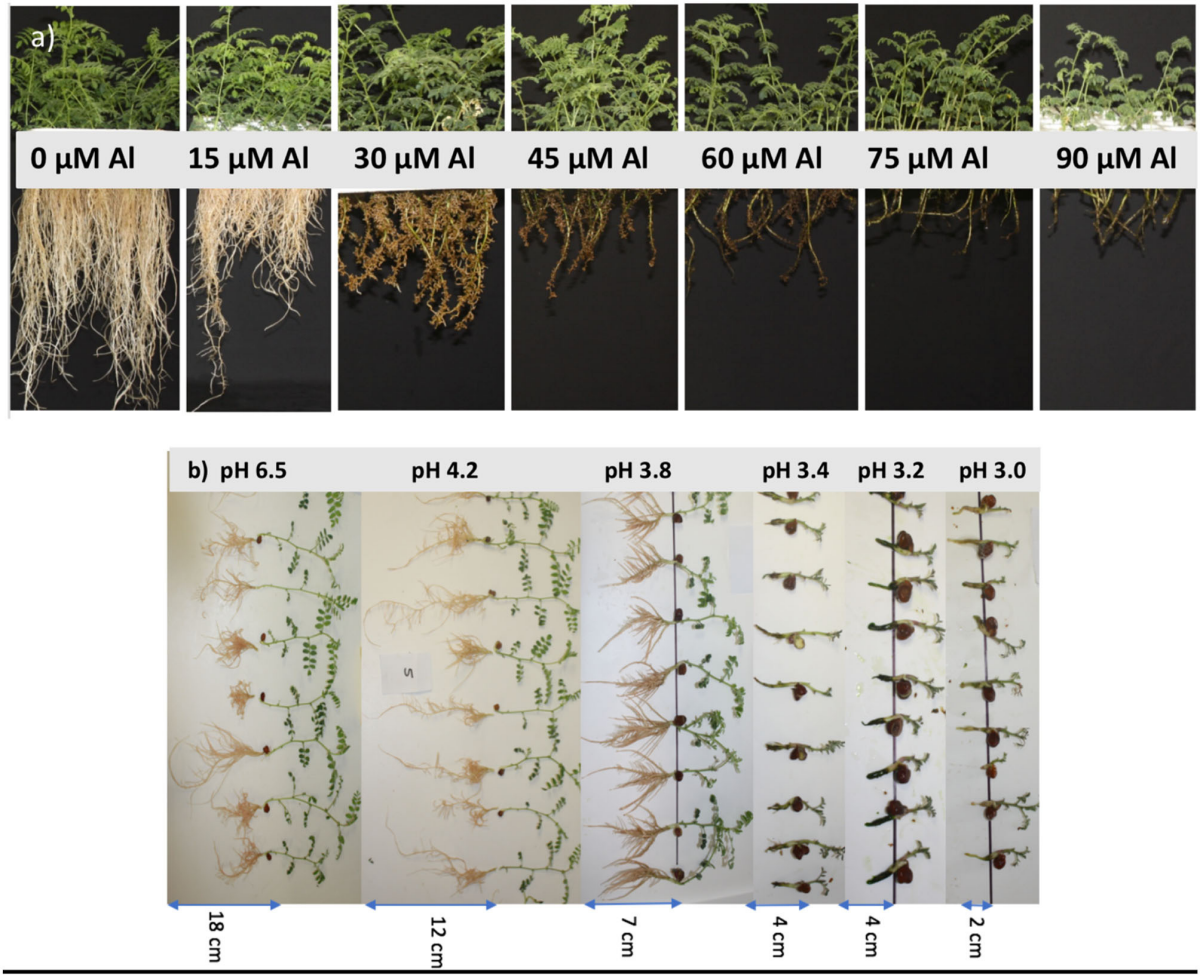
Plant growth of *Cicer arietinum* L. cultivars after 14 days in solution culture at **(a)** pH 4.2 and 7 Al levels (Experiment 2), and **(b)** pH 3.0, 3.2, 3.4, 3.8, 4.2, and 6.5 (Experiment 1).

The symptoms of Al toxicity were expressed as decreased root length and minimal elongation of lateral roots, which were short, stubby, and brown coloured. At 60 μM of Al and above, there was very little development of lateral roots (Figure 1a). Shoot growth was restricted in all experiments with Al added to solution. However, there were no apparent leaf symptoms during the experiments. Low pH of 3.0, 3.2, and 3.4, in the absence of Al, reduced shoot and root growth significantly; the roots were dark and short, and no lateral roots developed (Figure 1b).

### Experiment 1—Dose Response to pH

**Figure 2:**
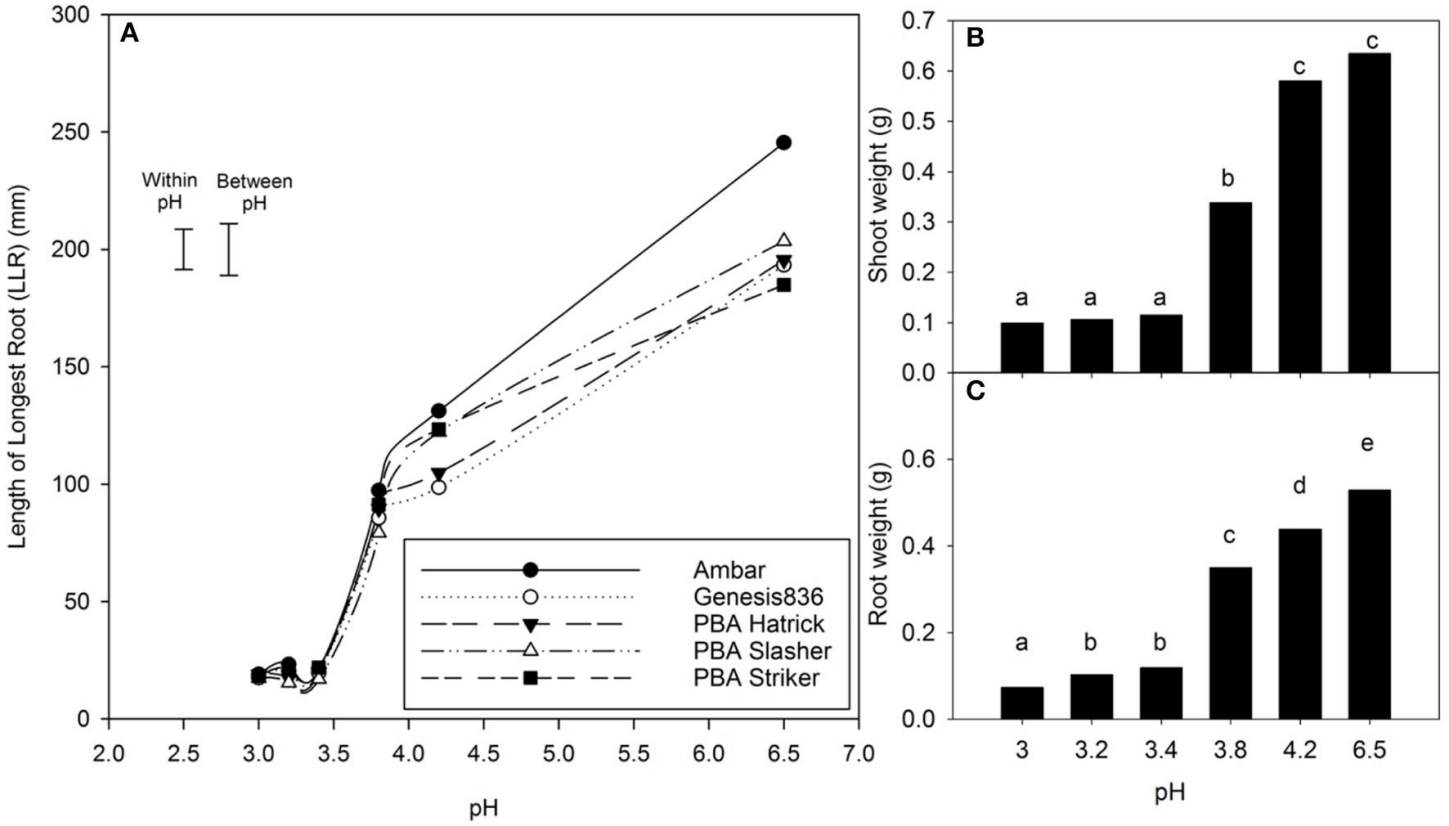
The **(A)** mean length of the longest root (LLR) and mean, **(B)** shoot weight, and **(C)** root weight of five cultivars of *Cicer arietinum* L. at the pH range from 3.0 to 6.5 in solution culture. Error bars are the least significant difference (LSD) at *P* = 0.05 for pH x cultivar either within pH treatments or between pH treatments. Data presented are back transformed means for total root and shoot weight (10 plants). Mean values with identical letters are not significantly different (Experiment 1).

There was a 44% reduction in mean LLR of *C. arietinum* L. cultivars in a solution of pH 4.2, 57% at pH 3.8, and 90% for pH 3.4 compared with the control (pH 6.5) (Figure 2). There was an interaction between treatment pH and cultivar for LLR (*P* < 0.001); at pH 4.2, Ambar, PBA Slasher, and PBA Striker had a greater LLR than Genesis 836 and PBA HatTrick. At a pH of 6.5, Ambar had a significantly greater LLR than all other cultivars. For each cultivar, there were differences in LLR with pH (*P* < 0.001), with a decrease in pH from 6.5 to 4.2 to 3.8. While each decrease in pH caused a significant decrease in LLR of cultivars Ambar, PBA Striker, and PBA Slasher, PBA HatTrick and Genesis 836 had no difference between pH of 4.2 and 3.8. In the pH range from 3.0 to 3.4, there was no difference in LLR with cultivar or pH treatments.

There was no interaction between treatment pH and cultivar for either shoot or root weight of *C. arietinum* L. cultivars. Shoot weight and root weight both declined with decrease in pH (*P* < 0.001) (Figure 2). The mean shoot weight of the plants at pH 3.4 and below was significantly less than the higher pH treatments, while the mean root weight was less with a pH of 4.2 compared with 6.5, whereas respective mean shoot weights were not different.

### Experiment 2—Dose Response to pH and Aluminium

**Figure 3:**
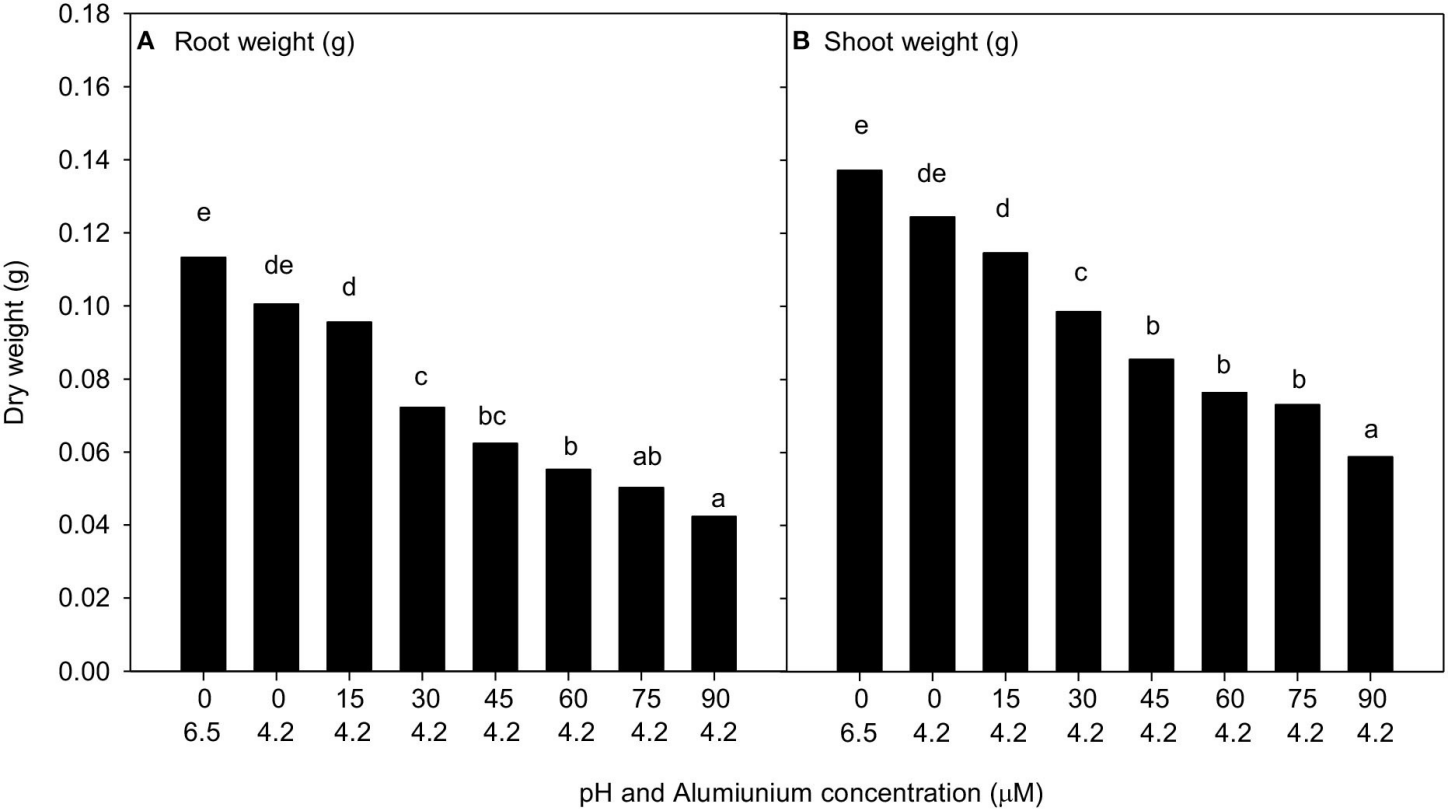
The mean **(A)** root weight and **(B)** shoot weight (g per plant) of all five *Cicer arietinum* L. cultivars in solution culture of pH 6.5 with 0 μM Al and pH 4.2 with Al 0 to 90 μM. Data presented are back transformed means for root and shoot weight per plant. Mean values with identical letters are not significantly different (Experiment 2).

There was no interaction between treatment and cultivar when *C. arietinum* L. was subjected to pH 6.5 without Al, pH 4.2 without Al, and six treatments at pH 4.2 and with Al concentrations between 15 and 90 μM. The mean shoot and root dry weights measured did not show any significant difference between pH 6.5 or 4.2 with 0 μM of Al. There were significant decreases in root and shoot weight with increasing Al concentration at pH of 4.2 from 15 to 90 μM (Figure 3) (*P* < 0.001); however, there was no difference among the responses of the five cultivars analysed. The lowest Al concentration that depressed root and shoot growth was 30 μM Al.

### Experiment 3—Dose Response to pH of 4.2 and Aluminium

**Figure 4:**
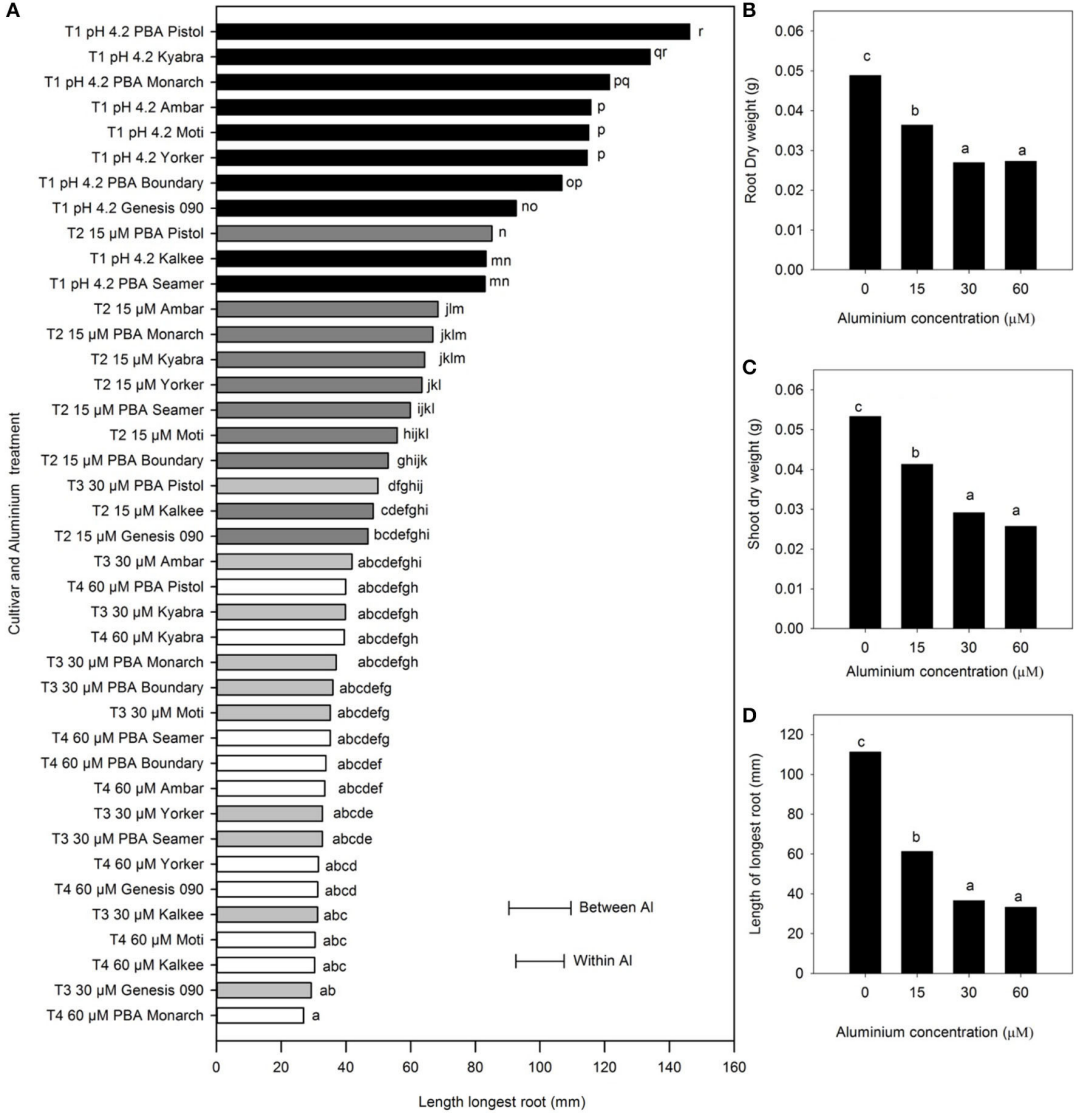
The **(A)** length of the longest root (LLR) of *Cicer arietinum* L. cultivars in solution culture, and **(B)** mean root dry weight (g), **(C)** mean shoot dry weight (g), and **(D)** mean length of longest root (mm) at pH 4.2 with 0, 15, 30, and 60 μM Al. Error bars are the LSD at *P* = 0.05 for Al treatment x cultivar either within aluminium treatment or between aluminium treatments. Means with identical letters are not significantly different. Shaded bars indicate aluminium concentration: T1 0 μM Al, black; T2 15 μM Al, dark grey; T3 30 μM Al, light grey; and T4 60 μM Al, white (Experiment 3).

The nine *C. arietinum* L. cultivars showed a significant decrease in mean root and shoot weights and LLR, with increase in Al concentration from 0 to 30 μM Al (*P* < 0.001); however, there was no difference between the 30 and 60 μM Al. At 15 μM Al, the relative reduction in shoot and root dry weight was 25%, while at 30 and 60 μM Al, it was 50%. Similarly, the LLR decreased for all cultivars (Figure 4). Among the cultivars, PBA Pistol had greater LLR at 15 μM Al than others and was similar to the LLR of some of the cultivars with no Al (*P* < 0.001). PBA Pistol consistently had the highest LLR, followed by Kyabra, PBA Monarch, and Ambar (a 0–30 μM Al range), while the sensitive cultivars in terms of LLR measurements were Genesis 090, Kalkee, and PBA Seamer.

### Experiments 4 and 5: Wild and Domesticated *Cicer* Screen

**Figure 5:**
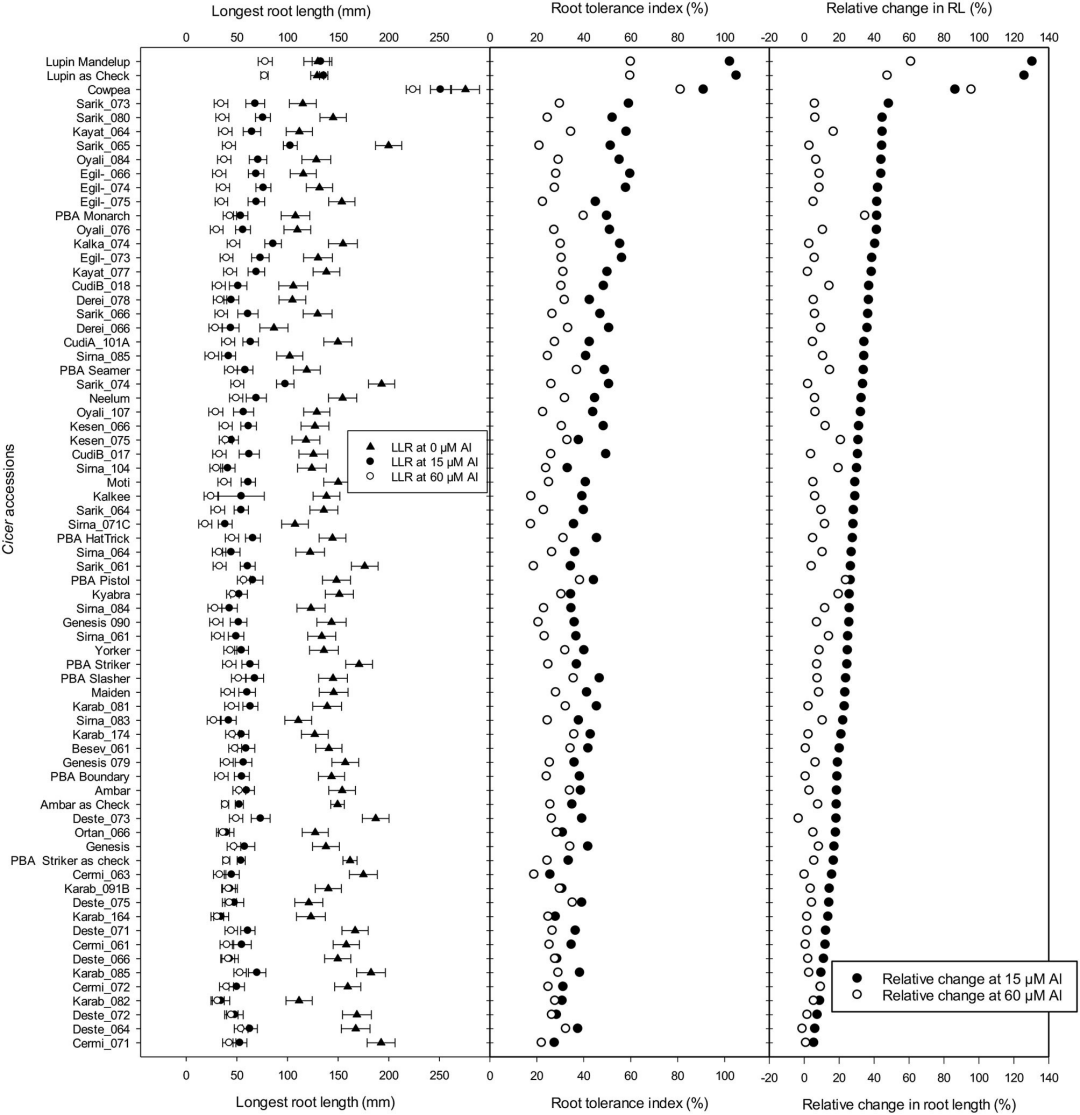
Length of longest root (mm), root tolerance index (RTI) (%), and relative change in root length (RRL) (%) of 49 wild *Cicer* accessions, 17 domestic cultivars, and Ambar, PBA Striker, lupin, and cowpea as checks at 0, 15, and 60 μM Al screened in Experiment 4. See Table 1 for the species classification and more information on accessions screened.

The longest root length measured after 10 days showed a significant reduction with Al treatment additions, and the LLR and RTI calculated for all accessions were ranked based on relative tolerance in accessions for change in root length at 15 μM Al (Figure 5). In Experiment 4, screened with 49 wild accessions and 17 domestic cultivars, the RTI of 13 wild *Cicer* accessions grown at 15 μM Al was higher than the most tolerant domestic chickpeas, PBA Monarch (Figure 5); they had RTI of ≥ 50% at 15 μM Al. All of the 13 most tolerant accessions were *C. retic* species belonging to population groups, Ret_5 and Ret_6 collected from Diyarbakir and Mardin Province, respectively, with exception of Oyali_84 and Oyali_76 from Ret_1 group collected from Adiyaman Province. On the other hand, the eight most sensitive accessions were from *C. echino* species for 15 μM Al treatment with RTI of ≤ 30%. However, at high Al of 60 μM Al, the most tolerant three were domestic cultivars, and no wild accessions were better than PBA Monarch with RTI of 40%. PBA Pistol and PBA Seamer had RTI of 38 and 37%, respectively, at 60 μM Al. Among the wild accessions, the RTI of two *C. echino* species from Urfa Province were high at 60 μM Al with RTI of 35%. In contrast to 15 μM Al, at 60 μM Al, most of the sensitive species were *C. retic* compared with *C. echino* and domestic species, *C. arietinium* L.

The results of change in RL, shoot and root dry weights of Experiment 4 are presented in Supplementary Figure B. Eight of the *C. retic* accessions ranked better than PBA Monarch, and they had RRL of ≥40% (Figure 5). Similar to RTI, for the change in root length, the tolerant accessions belong to the genetic population groups, Ret_5 and Ret_6. At 15 μM Al, the 10 most sensitive species were *C. echino* species with RRL of 15% and less. At 60 μM Al, PBA Monarch had high RTI of 35%, followed by PBA Pistol. Among the wild accessions, similar to 15 μM Al, most of the tolerant accessions were *C. retic* species, and sensitive were *C. echino* species.

The RSG and RRG derived for accessions are presented in Supplementary Figure C. The mean RSG of *Cicer* accessions was 72% (a range from 52 to 92%) and 44% (a range between 19 and 69%) at 15 and 60 μM Al, respectively. At 15 μM Al, RSG of domestic species was higher than wild species; six out of the top 10 tolerant accessions with RSG of ≥ 85% were domestic cultivars (Supplementary Figure C). Among the wild species, *C. retic* had higher mean RSG than *C. echino* species; *C. retic* accessions had 11% more RSG than *C. echino* at both 15 and 60 μM Al. At 15 μM Al, RRG ranged between 55 and 88%, with *C. arietinium* L. and *C. retic* accessions having higher RRG than *C. echino* accessions. Among the *Cicer* accessions, domestic cultivars Maiden and PBA Seamer had high RSG and RRG at 15 μM Al. At 60 μM Al, RRG ranged between 29 and 65%, and the five most tolerant lines in terms of high relative shoot and root growth were domestic *C. arietinium* L.

The results of Experiment 5 were analysed based on their genetic population groups and also individual accessions. The accessions belong to 13 population groups, 8 were from *C. retic* and 5 from *C. echino* groups (Table 1). Due to a large number of accessions (118 + checks) screened, the results of genetic population groups are presented here. Moreover, the accessions did not show a significant difference within the population group for the parameters measured and the results of individual accessions fitted in the model explained above are presented in Supplementary Figures D–F.

**Figure 6:**
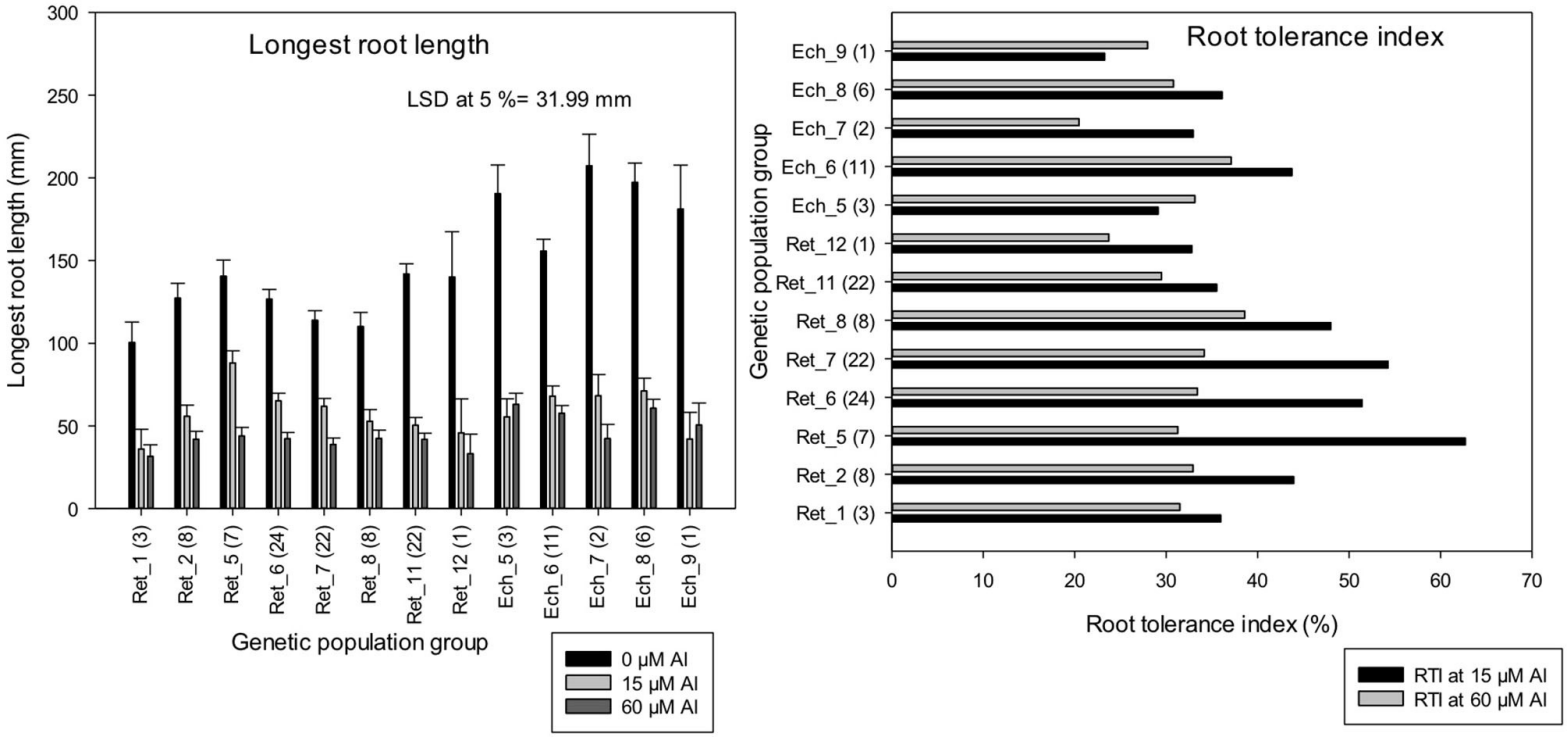
Length of longest root (mm) and root tolerance index (RTI) (%) of 118 wild *Cicer* accessions under 13 genetic population groups at 0, 15, and 60 μM Al screened in Experiment 5. The number in parentheses represents the number of accessions screened for a group. See Table 1 for the species classification and more information on accessions screened.

The LLR of *C. retic* groups, Ret_1, Ret_7, and Ret_8 measured at harvest of control (0 μM Al) was significantly lower than all of the *C. echino* groups (at LSD, 5%). However, Al treatments, 15 and 60 μM Al did not show significant difference among most of the *C. retic* and *C. echino* groups. The genetic populations, Ret_5 (from Diyarbakir Province), Ret_6 and Ret_7 (Mardin Province) had >50% RTI at 15 μM Al (Figure 6). Similar greater tolerance to Al toxicity was seen in groups Ret_5 and Ret_6 in previous screening in Experiment 4. Among the wild *C. retic* accessions screened in Experiment 5, Ret_5 had a high RTI of 63% at 15 μM Al, and Ret_8 from Mardin had a high RTI of 39% at 60 μM Al, whereas, Ret_12 collections from Sirnak Province had low RTI of 33 and 23% at 15 and 60 μM Al, respectively. Among *C. echino* accessions, the population group, Ech_6 from Siv-Diyar collection site of Urfa Province, had a high RTI of 43 and 37% at 15 and 60 μM Al, respectively, and Ech_5 from Destek collection site of Urfa Province had lowest RTI of 23%. The mean RTI calculated for *C. retic* species based on genetic populations was 12% higher than *C. echino* populations at 15 μM Al.

**Figure 7:**
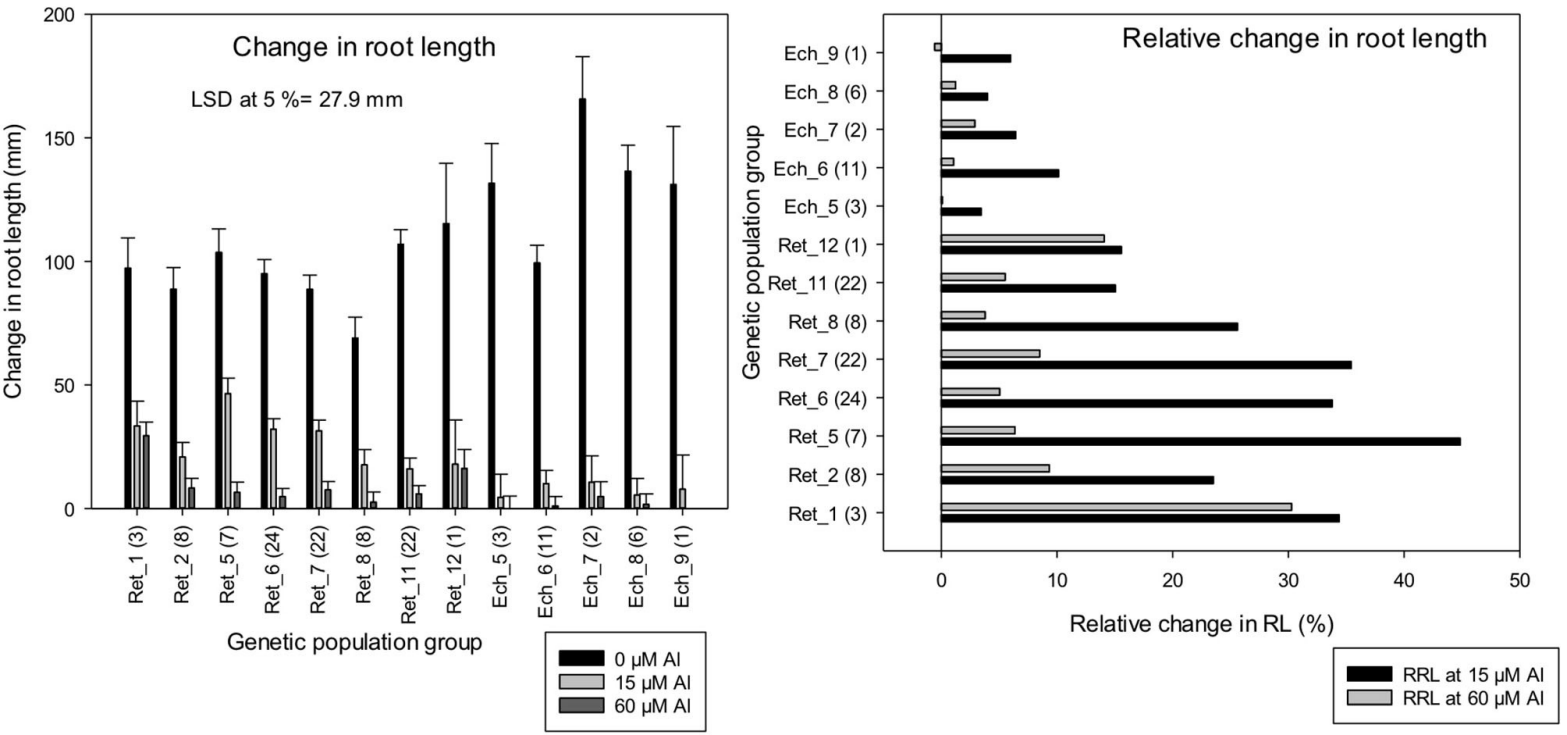
Change in root length (mm) and relative change in root length (RRL) (%) of 118 wild *Cicer* accessions under 13 genetic population groups at 0, 15, and 60 μM Al screened in Experiment 5. The number in parentheses represents the number of accessions screened for a group. See Table 1 for the species classification and more information on accessions screened.

Consistent with LLR, change in RL measurements in the control solution was higher in *C. echino* groups, Ech_7 and Ech_8, which had a significantly higher change in RL than all of the *C. retic* groups (Figure 7). However, at 15 and 60 μM Al, Ret_5 and Ret_1 groups had a significantly higher change in RL measurements, respectively, than all of the *C. echino* groups (at LSD, 5%). The LLR measurements and the change in RL measured for the accessions are well-correlated (*r* = 0.95). Relative change in root length in population groups, Ret_5, Ret_6, Ret_7, and Ret_1 (>30%) was higher than Ret_11 and Ret_12, and all of the *C. echino* groups. Ret_5 had a significantly high RRL of 44% at 15 μM Al, compared with Ret_11 and Ret_12, which had only 15%. Interestingly, Ret_1 group maintained high RRL both at 15 and 60 μM Al, with 34 and 30%, respectively. Similar to RTI, *C. echino* species showed smaller changes between population groups when compared with *C. retic* populations. Unlike RTI, the RRL showed significant discrimination between *C. retic* and *C. echino* species. The mean RRL calculated for *C. retic* population groups at 15 μM Al was (28.5%) significantly higher than *C. echino* species (6%).

**Figure 8:**
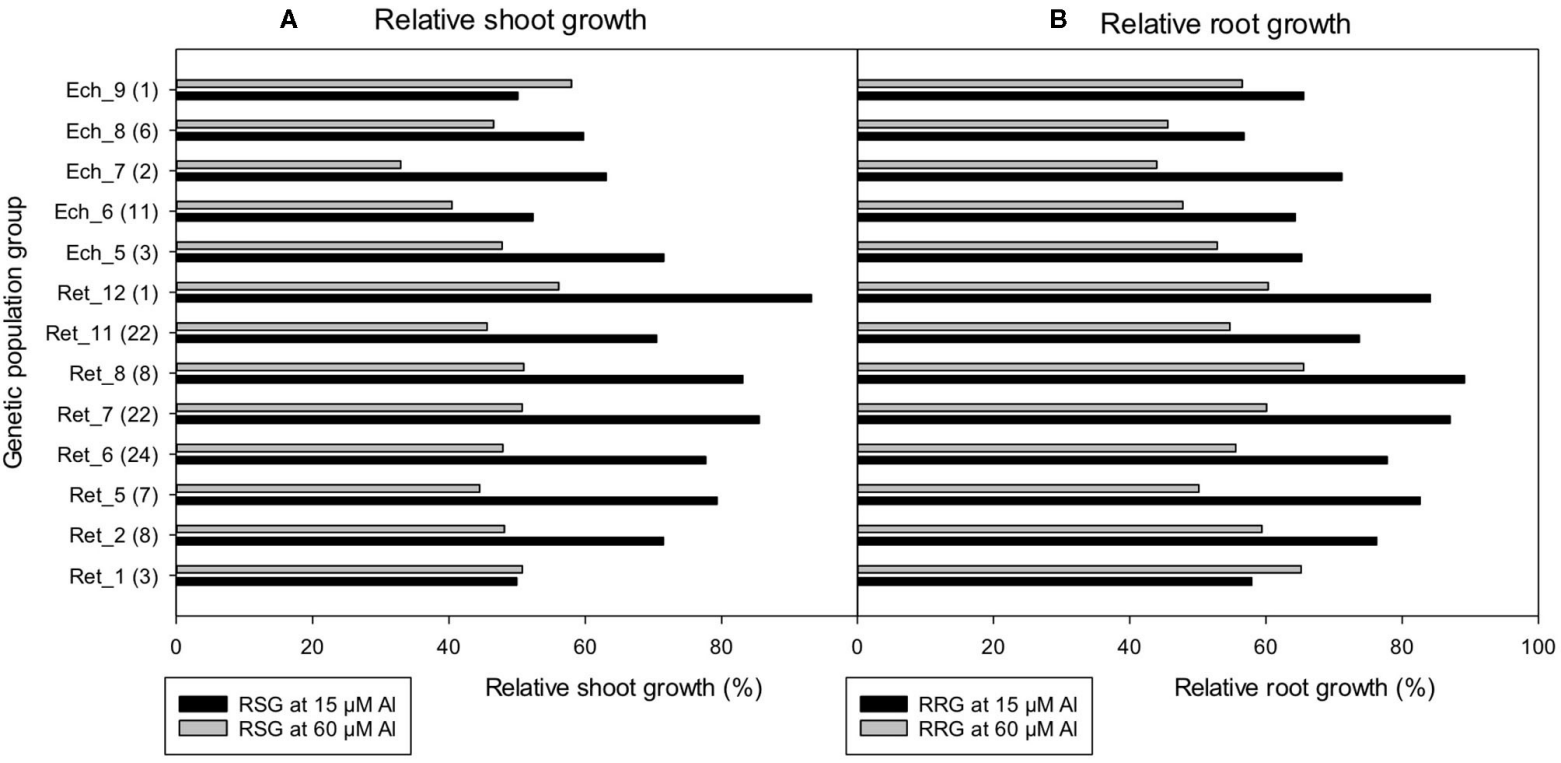
The **(A)** relative shoot growth (RSG) (%) and **(B)** relative root growth (RRG) (%) of 118 wild *Cicer* accessions under 13 genetic population groups at 0, 15, and 60 μM Al screened in Experiment 5. The number in parentheses represents the number of accessions screened for a group. See Table 1 for the species classification and more information on accessions screened.

In contrast to root length measurements, shoot and root dry weight showed less difference among the population groups. In general, the mean relative shoot and root dry weights of *C. retic* groups were higher than *C. echino* by 17 and 14%, respectively. There was a significant correlation between shoot and root dry weights measured in the population groups (*r* = 0.92). Relative shoot and root growth were less sensitive than root length measurements, even with high Al treatment of 60 μM Al where wild *Cicer* maintained >40% RSG and RRG, except for Ech_7 group that had 33% RSG (Figure 8).

The RRL was correlated significantly with RTI (*r* = 0.725), RSG (*r* = 0.590), and RRG (*r* = 0.694). Among the parameters measured, the RRL was more sensitive to Al treatments and discriminated among the population groups of accessions better than other measured parameters. Moreover, since the change in root length is the net change in root length, this measure eliminates any initial differences in plant growth, and, hence, the relationship between different *Cicer* species for mean change in RRL was computed. In general, the large scale *Cicer* screening of germplasm (Experiments 4 and 5) showed the wild species *C. echino* was more sensitive to Al treatments than *C. retic* and domestic species, *C. arietinium* L. The RRL of accessions screened from *C. echino* showed a significant reduction with Al treatments when compared with *C. retic* in Experiment 5 (Supplementary Figure F); the mean RRL of *C. echino* accessions at 15 and 60 μM Al treatments was 12 and 2%, respectively, whereas those of *C. retic* were 26 and 7%, respectively.

### Experiment 6: Confirmation Screening

**Figure 9:**
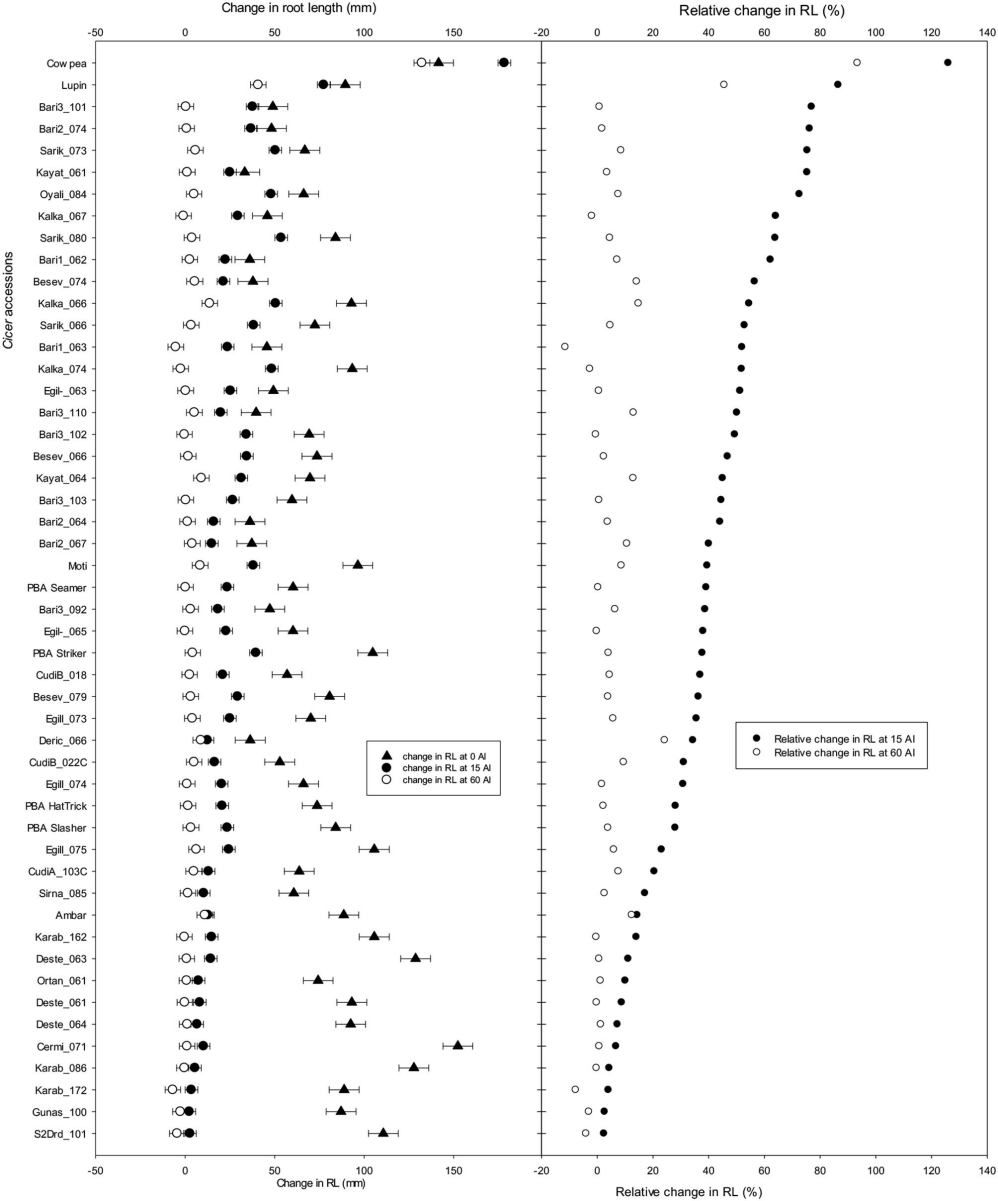
Change in root length (mm) and relative change in root length (%) (RRL) of 42 wild *Cicer* accessions, 6 domestic cultivars, cowpea and lupin as checks grown in solution with pH 4.2 and 0, 15, and 60 μM Al. See Table 1 for the species classification and more information on accessions screened (Experiment 6).

A selection of 42 wild accessions and 6 domestic cultivars from large scale screening experiments, depending on seed availability, was used in the confirmation screening and compared with the check species to assess the consistency of their ranking for Al tolerance. From the results of Experiments 4 and 5, accessions which had >40% and <20% RRL were grouped as tolerant or sensitive to Al toxicity, respectively. From each experiment, 10 accessions classified as tolerant, 10 accessions classified as sensitive, and a few random accessions were selected and screened. The LLR, RSG, and RRG are presented in Supplementary Figure G. The RRL ranged between 2 and 75% at 15 μM Al (Figure 9). The combined results of RRL percentages ranks from Experiments 4 and 5 were compared with the confirmation screening. At 15 μM Al, 6 out of 10 most tolerant accessions with high RRL in the confirmation screens were in the top 20 list of accessions with high RRL in Experiments 4 and 5 combined ranking, and all 10 of the sensitive accessions with low RRL in confirmation screens were among the 20 worst performing accessions in the combined (Experiments 4 and 5) ranking.

## Discussion

The composition of solutions used to screen Al toxicity at low pH is critically important since many factors can alter Al speciation and Al^3+^ activity in solution. Previous studies in chickpeas used high strength Hoagland solution (Choudhury and Sharma, 2014) or different compositions for nutrient solutions (Rai, 1991; Singh and Raje, 2011; Sharma et al., 2015), making it difficult to compare chickpea responses to low pH and Al. The Al^3+^ species is the main form of Al toxic to plants at low pH and a dominant form of Al found in solutions, but it can form hydroxyl monomers of Al and also complexes or precipitates with other ions, which means that some Al in solution is no longer toxic to plant growth (Kochian et al., 2005; Famoso et al., 2010). Previous research suggests that the proportion of added Al that remains as soluble Al^3+^ is greater and more toxic when the solution reflects the concentrations of soluble ions and the ionic strength of soil solutions, and has low total ionic strength; low concentrations of most nutrients; controlled ratios of NH_4_ to NO_3_; and low concentration of P (Blamey et al., 1991; Kopittke et al., 2010). In the present study, the solution composition recommendations by Blamey et al. (1991) and Kopittke and Blamey (2016) were followed, leading to solution pH ≤ 4.5 and phosphorus ≤ 5 μM, respectively. The ionic concentration was low, ranging between 2,820 and 3,290 μM, and the solution was changed every 2 days to ensure deficiency of required nutrients did not confound the experiment, and that the Al in solution remained close to the treatment concentration. The use of Geochem-EZ showed that the solution composition was optimised to maintain a complete nutrient solution for plant growth, with minimal loss of Al due to complexes forming with metals and ligands in the solution. Also, the model confirmed that the free Al^3+^ in solution increased by decreasing the PO_4_ from 13 μM in earlier dose response experiments to 5 μM as recommended by Kopittke and Blamey (2016).

For *C. arietinum* L. cultivars, 15 μM Al inhibited root elongation due to Al toxicity, 30 μM Al restricted lateral root development while 60 μM Al severely restricted root length and lateral root development. The 15 and 60 μM Al concentrations were selected to be used in large scale screening of *Cicer* germplasm with the hypothesis that, at 15 μM Al, tolerant accessions would be able to maintain plant growth similar to control, and, at 60 μM Al, accessions that had increased root length or lateral root development would have a greater level of tolerance than the remainder of the collection. Aluminium concentrations selected in this study (15 and 60 μM Al) were lower than the concentrations of other researchers, Choudhury and Sharma (2014), Rai (1991), Singh and Raje (2011), which used concentrations, 10–500, 741, and 100 μM Al, respectively. However, these screening solutions (Rai, 1991; Singh and Raje, 2011; Sharma et al., 2015) had high ionic strength, which likely caused the complexing of Al with other nutrients in the solution such as sulphate or phosphate. Therefore, the concentrations of Al reported probably exceeded the Al^3+^ that was actually present in the solution (Kochian et al., 2005; Shaff et al., 2010; Shavrukov et al., 2012).

The LLR and the RRL were more sensitive to changes in pH than plant biomass parameters as evident from the effects of a decrease in solution pH from 6.5 to 4.2. Similarly, with the addition of Al, the root length was more responsive to solution Al toxicity than plant biomass parameters. Root growth inhibition is the primary and earliest symptom of Al toxicity; hence, root growth is used extensively in screening studies. The RRL has served as a marker for Al toxicity and identification of tolerance capacity in plants (Awasthi et al., 2017). The response of root length to Al stress has been used to screen rice (*Oryza sativa)*, sorghum (*Sorghum bicolor)*, wheat (*Triticum aestivum*) (Rout et al., 2001), and other pulses like soybean (*Glycine max*) (Horst and Klotz, 1990), lentil (*Lens culinaris* Medikussubsp. *culinaris*) (Kulkarni et al., 2021), chickpeas, pigeon peas (*Cajanuscajan L*. Millsp.) (Choudhary et al., 2018) and other temperate legume genotypes (Rout et al., 2001). According to previous research in chickpeas, under Al stress root growth was primarily inhibited, and it was attributed to the production of excess H_2_O_2_, which can result in disruption of cellular redox balance and the inactivation of an antioxidant defence mechanism, and there is loss of plasma membrane integrity under excess Al^3+^ (Choudhury and Sharma, 2014).

Genotypic variation within *C. arietinum* L. cultivars for Al toxicity tolerance was reported in other studies (Rai, 1991; Singh and Raje, 2011). Similarly, in the present study, among the Australian domestic cultivars, PBA Pistol and PBA Seamer had better root growth in low pH with Al solutions than other chickpea cultivars. In the dose response study (Experiment 3), PBA Pistol consistently had the longest LLR at all Al concentrations (15–60 μM) and had no difference in LLR between concentrations of 0 and 15 μM Al. However, in the large-scale screening with wild *Cicer* (Experiment 4), PBA Pistol was not the best performing cultivar, but it had reasonable RRL of 26 and 24% at 15 and 60 μM Al, respectively. Interestingly, PBA Seamer did not show any difference in tolerance during the dose response experiment compared with the other cultivars; however, it was one of the best performing chickpea cultivars in the screening with wild *Cicer* at 15 μM Al (Experiments 5 and 6), with RRL of 34 and 39%. Also, PBA Monarch showed better Al tolerance than other cultivars in Experiment 5 with RRL of 42 and 35% at 15 and 60 μM Al, respectively. Hence, among chickpea cultivars, their apparent Al toxicity tolerance lacked consistency among studies. Nevertheless, all screening experiments with chickpeas confirmed that their Al toxicity tolerance was markedly less than lupin and cowpea, two legumes with known Al toxicity tolerance (Howeler, 1991; Choudhury and Sharma, 2014).

This is the first study to screen a wide collection of wild *Cicer* species, 127 and 40 from species *C. retic* and *C. echino*, respectively, collected from five provinces in Turkey. Even though these accessions originated from a narrow geographical area, they had genetic differentiation due to a range of climate, soil, and elevation (ranging between 740 and 1,695 m) among collection sites (von Wettberg et al., 2018). Moreover, *C. retic* species were found to occur at higher elevations than *C. echino* and were found on soils developed from limestone and sandstone, whereas, *C. echino* was from soils developed from basalt (von Wettberg et al., 2018), suggesting different edaphic requirements for the two species (Reen et al., 2019). Moreover, *C. retic* collection sites were more fertile and alkaline than *C. echino* sites (von Wettberg et al., 2018); five core samples analysed from each of the collection sites showed that the Ca, K, and Mg concentrations (g/kg), and total organic carbon (%) in *C. retic* collection sites were nearly twice as that of *C. echino* collection sites. The pH, EC (μS/cm), organic matter (%) of *C. retic*, and *C. echino* sites were 7.64, 333 and 6.45%, and 7.23, 335 and 5.55%, respectively. The soil concentrations of P, Zn, Fe, Mn, Cu, and Na were higher in *C. echino* sites than *C. retic* sites. In the present study, with 167 wild *Cicers*, in general, *C. retic* had greater tolerance to Al toxicity at 15 μM Al with high RTI, RRL, RSG, and RRG compared with *C. echino*, with RRL showing better discrimination between the species than relative plant dry weights.

In this large-scale screening, none of the *C. echino* accessions had RRL of >40%, and only one of the *C. arietinum* L. (cultivar: PBA Monarch) had >40%. However, 19 *C. retic* accessions had better Al toxicity tolerance with RRL of ≥40% at 15 μM Al. Nevertheless, Al tolerance was not maintained in these accessions at 60 μM Al. Only six of the accessions had RRL of ≥20% at 60 μM Al; two domestic cultivars, PBA Monarch and PBA Pistol, and four of the *C. retic* accessions.

In acid soils with P deficiency, understanding the mechanisms relating to interactions between Al and P in chickpeas will facilitate the development of more Al-tolerant cultivars. Previous research on P acquisition showed genotypic variation among chickpea genotypes in root growth parameters and root carboxylate exudation, particularly malonate (Pang et al., 2018). The root organic acid exudation in many plant species was found to be the main mechanism that can solubilise soil P and modify soil properties in acidic/Al toxic soils where P fixation is an issue (Liao et al., 2006). In Al-tolerant species and cultivars, high levels of organic acid secretion, as malate, citrate, or oxalate, help to chelate or detoxify Al and prevent Al from interacting with root apices (Foy et al., 1978; Bian et al., 2013). In this screening, if organic acid excretion is the main Al toxicity tolerance mechanism, the level of expression was insufficient to provide tolerance to severe Al toxicity. Screening of accessions in high Al concentrations was effective in identifying barley lines tolerant to a wide range of acid soils. In the barley screening, concentrations of 8, 20, and 100 μM Al were used in different experiments to confirm Al toxicity tolerance (Dai et al., 2011). Similar research of 300 barley accessions from eight genetic population groups (collected worldwide) identified new acid tolerant lines which outyielded the current Australian barley cultivars by >20% in acid soils and >30–90% in extremely acid soils (Li, 2016). However, in this *Cicer* screening, there is lack of consistency in tolerance between 15 and 60 μM Al, and, moreover, it is evident that none of the 187 accessions belonging to the three *Cicer* species showed tolerance comparable to cowpea or lupin. Therefore, the search for greater Al toxicity tolerance and low pH in *Cicer* should continue, especially incorporating all the novel wild collections and landraces, particularly from parts of the world with known acid soils, which may be the key for future breeding programs in *Cicer*, targeting acid soil tolerance.

Predominantly, the genetic population groups were linked to the site of origin by von Wettberg et al. (2018), and, in this study, among the wild *C. retic* group, Ret_ 5 from Egil and Kalkan collection sites showed greater tolerance to Al toxicity and Ret_11 from CudiA and CudiB collection sites showed high sensitivity to Al toxicity at 15 μM Al. Interestingly, the sensitive collection sites CudiA and CudiB were from high elevation of 1,286 and 1,366 m, respectively when compared to Al tolerant sites, Egil and Kalkan from elevation of 987 and 841 m, respectively. The differences among *C. echino* population groups were small compared with *C. retic*, which may be attributed to relatively homogeneous soils derived from basaltic rocks where they were collected and also reflecting a much narrower range of environments where the species occurs. Also, *C. echino* had lower number of polymorphic loci than *C. retic* (88,976 vs. 136,638), showing less genetic diversity than *C. retic* (von Wettberg et al., 2018).

In strongly acidic soils, soluble Al and Mn are frequently both excessive and affect crop production. In such soils where free Al^3+^ is more than 0.2 cmol/kg soil and exchangeable Mn^2+^ concentration reaches 2–9 cmol/kg, soil Al and Mn toxicity is considered highly problematic to plant growth (Yang et al., 2009). The interactions between these two elements were either antagonistic or synergistic, depending on their concentrations and specific physiological response. In plant species like soybeans (Yang et al., 2009), wheat (Blair and Taylor, 1997) and cowpea (Taylor et al., 1998), excess Al had antagonistic effect on Mn uptake and alleviated Mn toxicity symptoms. In chickpeas, *C. retic*, which had greater Al tolerance than *C. echino*, also had increased Mn uptake in plant tissues (Sultana et al., 2020), and it has been hypothesised this was due to competition between Al^3+^ and Mn^2+^ for the binding sites of cell wall or plasma membrane (Yang et al., 2009). Also, in a recent Mn screening study in *Cicer*, the Mn toxicity tolerance ranking contrasted with the current Al toxicity study, since the *C. echino* accession was more tolerant to Mn than the *C. retic* accession, but, there was only one accession in each of wild *Cicer* examined for Mn toxicity (Pradeep et al., 2020).

Variation in response to Al toxicity in plant species between hydroponic screening and soil screening has been reported due to difference in responses of root length (Shavrukov et al., 2012), root hair density (Genc et al., 2007), and a root growth pattern (Moroni et al., 2010). In general, correlation among assays is good for a common set of genotypes; however, previous screening in barley and wheat genotypes (Moroni et al., 2010) showed variable responses to Al toxicity between soil and a solution assay, which implies standardisation of screening protocols is an important component when breeding for Al toxicity. There have not been any comparable studies in *Cicer*, and validation of Al toxicity tolerance in Al-toxic acid soil is needed to confirm the Al tolerance of domestic and wild *Cicer* accessions, and also to avoid any misclassification of accessions for tolerance to Al toxicity between solution culture and an acidic soil assay.

There were only 18 and 10 original accessions of *C. retic* and *C. echino* in world gene banks previously, but recent collection missions have increased the collections manifold (Talip et al., 2018; von Wettberg et al., 2018). The expanded collection appears to show novel sources with tolerance to Al toxicity, which could be useful for developing an acid tolerant chickpea. In this screening, 15% of *C. retic* collections screened were found to be more Al tolerant than domestic cultivars. Moreover, this was the first study to evaluate the newly collected material for Al toxicity tolerance in low ionic nutrient solution and offers information for improving our understanding of this phenotype and adding genetic resources for the plant breeders to select from Al-tolerant (potentially acid soil tolerant) accessions and deploy germplasm that possibly includes combined abiotic and biotic resistance.

## Conclusions

The response of domesticated and wild *Cicer* growth was assessed in low ionic strength solution in the presence of soluble Al at pH 4.2. The dose response to solution Al indicated that root length and the root growth indices, root tolerance index, and relative root length are the best variables to differentiate between tolerant and sensitive accessions. Among the domestic cultivars, rankings for Al toxicity tolerance were inconsistent across experiments but always much inferior to lupin and cowpea, the check species. The wild *C. retic* species was found to be more tolerant than *C. echino*. The relative root length of 19 wild *C. retic* species was ≥ 40% with Al toxicity of 15 μM Al, and 13 *C. retic* accessions had higher root tolerance index than PBA Monarch. Among the *C. retic* accessions, the genetic population groups Ret_5, Ret_6, and Ret_7 from Diyarbakir and Mardin Province were more tolerant than other groups.

## Data Availability Statement

The original contributions presented in the study are included in the article/Supplementary Material, further inquiries can be directed to the corresponding author/s.

## Author Contributions

WV and SS designed and conducted the experiments under the supervision of RB. KP wrote the manuscript with revisions provided by RB and WV. SD developed the statistics model and performed analysis. All authors discussed the results and reviewed the manuscript.

## Conflict of Interest

SD is a Director of the company company Apex Biometry. The remaining authors declare that the research was conducted in the absence of any commercial or financial relationships that could be construed as a potential conflict of interest.
