# Supplementary material for: Novel Sources of Tolerance to Aluminium Toxicity in Wild Cicer (Cicer reticulatum and Cicer echinospermum) Collections
Source: Front Plant Sci. 2021 Jun 25;12:678211. doi: 10.3389/fpls.2021.678211 (PMC8269930; doi:10.3389/fpls.2021.678211)
Supplement: Supplementary file 1 [file Data_Sheet_1.docx]

**Appendix A**

**Novel sources of resistance to aluminium toxicity in wild *Cicer* (*Cicer reticulatum* and *Cicer echinospermum*) collections**

**Supplemetary data**

Figure A. Relationship between aluminium (Al) in the nutrient solution for a) and b) free Al^3+^ concentration and c) ionic strength (μM) Relationship between aluminium (Al) in the nutrient solution and free Al^3+^ concentration from the literature compared with our solution as calculated with GEOCHEM-EZ. In a) solutions shown up to 800 µM Al and in b) solutions shown up to 100 μM.

Table A Comparison of nutrient solutions of this study with previous studies by other researchers.

|  | Moroni *et al.* (2010) | Hede *et al.* (2001,2002) | This study | Sing and Raje (2011) | Choudury and Sharma (2014) |
| --- | --- | --- | --- | --- | --- |
| Crop | Barley | Cereals | Chickpea | Chickpea | Chickpea |
| pH | 4.3 | 4 | 4.2 | 4.5 | 4.7 |
| Ionic strength | 6074 | 2719 | 2820 | 26560 | 3102 |
| Ca | 1000 | 400 | 400 | 4000 | 100 |
| K | 1020 | 650 | 650 | 6500 |  |
| Mg | 400 | 250 | 250 | 2500 |  |
| NH_3_ | 600 | 60 | 50 | 600 |  |
| NO_3_ | 3400 | 690 | 690 | 6900 |  |
| Mn^2+^ | 9 |  | 9 |  |  |
| B(OH)_4_ | 23 |  | 23 |  |  |
| MoO4 | 0.1 |  | 0.1 |  |  |
| Zn | 0.8 |  | 0.8 |  |  |
| Cu^2+^ | 0.3 |  | 0.3 |  |  |
| SO_4_ | 441 | 10 | 21 | 100 |  |
| PO_4_ | 100 |  | 13 |  |  |
| Na | 40 |  | 66 |  |  |
| Cl | 78 | 1300 | 1378 | 13000 | 20 |
| Fe^3+^ | 20 |  | 20 |  |  |
| EDTA | 20 |  | 20 |  |  |


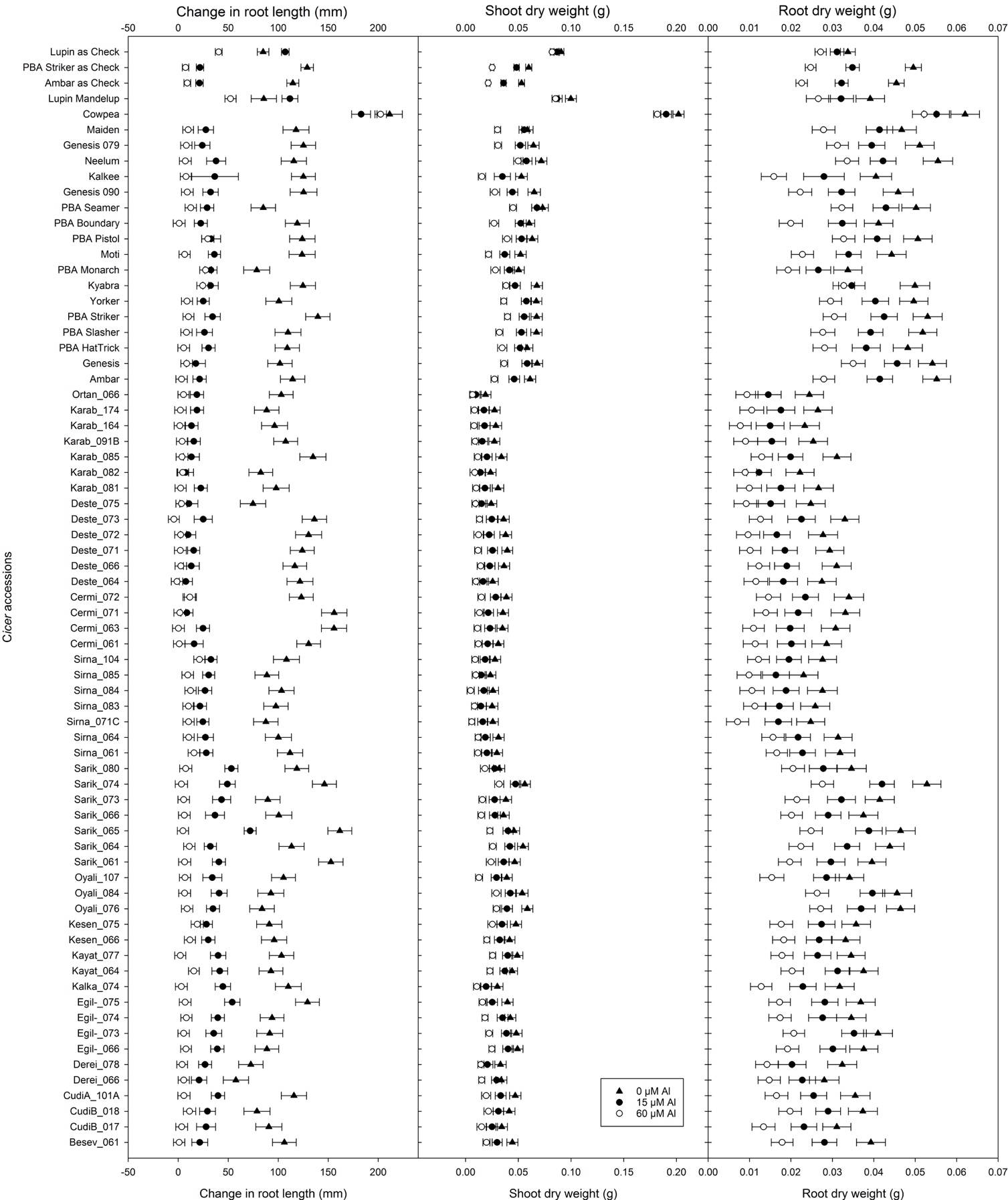


**Fig B** The change in root length (mm), shoot and root dry weight (g) of 49 wild *Cicer* accessions, 17 domestic cultivars, and Ambar, PBA Striker, lupin and cowpea as checks at 0, 15 and 60 μM Al screened in Experiment 4. See Table- 1 for the species classification and more information on accessions screened.

**Fig. C** Relative shoot growth (%) (RSG) and relative root growth (RRG) (%) of 49 wild *Cicer* accessions, 17 domestic cultivars, and Ambar, PBA Striker, lupin and cowpea as check at 0, 15 and 60 μM Al screened in Experiment 4. See Table 1 for the species classification and more information on accessions screened.

**Experiment 5**

The longest root length measured after 10 days showed a significant reduction with Al treatment additions, and the LLR and root tolerance index (RTI) percentages calculated for all accessions were ranked based on accessions having most tolerant RTI at 15 μM Al (Fig. D). The RTI of accessions screened at 15 μM Al ranged between 55 and 30 %, and at 60 μM Al between 44 and 22 %. In general, there were no difference between *C. retic* and *C. echino* species for RTI with Al treatments. The mean RTI of 94 *C. retic* accessions were 43 and 30.5 % at 15 and 60 μM Al, respectively, and that of the 23 *C. echino* accessions screened were 44 and 35 % at 15 and 60 μM Al, respectively. Based on the RTI, the 10 most tolerant accessions at 15 μM Al were *C. retic* species, however, at 60 μM Al, 6 of the top 10 were *C. echino* species.

The change in root length, shoot and root dry weights measured for accessions screened in Experiment 4b are presented as supplementary data, Fig. E. The relative change in root length (RRL), and relative shoot growth (RSG) and relative root growth (RRG) as dry weight percentages derived for the accessions were ranked based on the relative tolerance to sensitivity in accessions for change in root length at 15 µM Al (Fig. F). In general, accessions with low growth in 15 µM Al also had low growth in 60 µM Al for all growth parameters, however, there were some exceptions. Relative change in root length percentage (RRL) of *Cicer* species was correlated to the response of other growth parameters (RRG, RSG) and had the greater sensitivity to Al toxicity than other parameters like root tolerance index, and relative shoot and root dry weights measured.

The RRL in *Cicer* accessions showed a significant reduction with Al treatments, and the change in RRL ranged between 46 to 5 %, and 31 to -2.5% at 15 and 60 µM Al treatments, respectively (Fig. F). At 15 µM Al, the mean RRL of *C. retic* accessions was 26 %, whereas, that of *C. echino* accessions was only 12 %; the top 10 tolerant accessions were *C. retic* species, and 8 of the top 10 sensitive accessions were *C. echino* species for RRL at 15 µM Al. At high Al level of 60 µM, the mean RRL of *C. retic* accessions were 7 % and that of *C. echino* accessions were only 2 %. Similar to 15 µM Al, *C. retic* accessions was found to be tolerant than *C. echino* accessions at 60 µM Al.

The mean RSG of *Cicer* accessions at 15 and 60 µM Al were 71 and 46 %, respectively, and RRG were 74 and 55 % at 15 and 60 µM Al, respectively. Among the accessions screened, *C. retic* germplasm had 6 % more plant growth (RRG and RSG) than *C. echino* at both 15 and 60 µM Al. Similar to RRL, *C. retic* accessions showed better tolerance to Al treatments than *C. echino* with plant growth, the top 10 tolerant accessions ranked for RSG and RRG in Experiment 4a were *C. retic*.


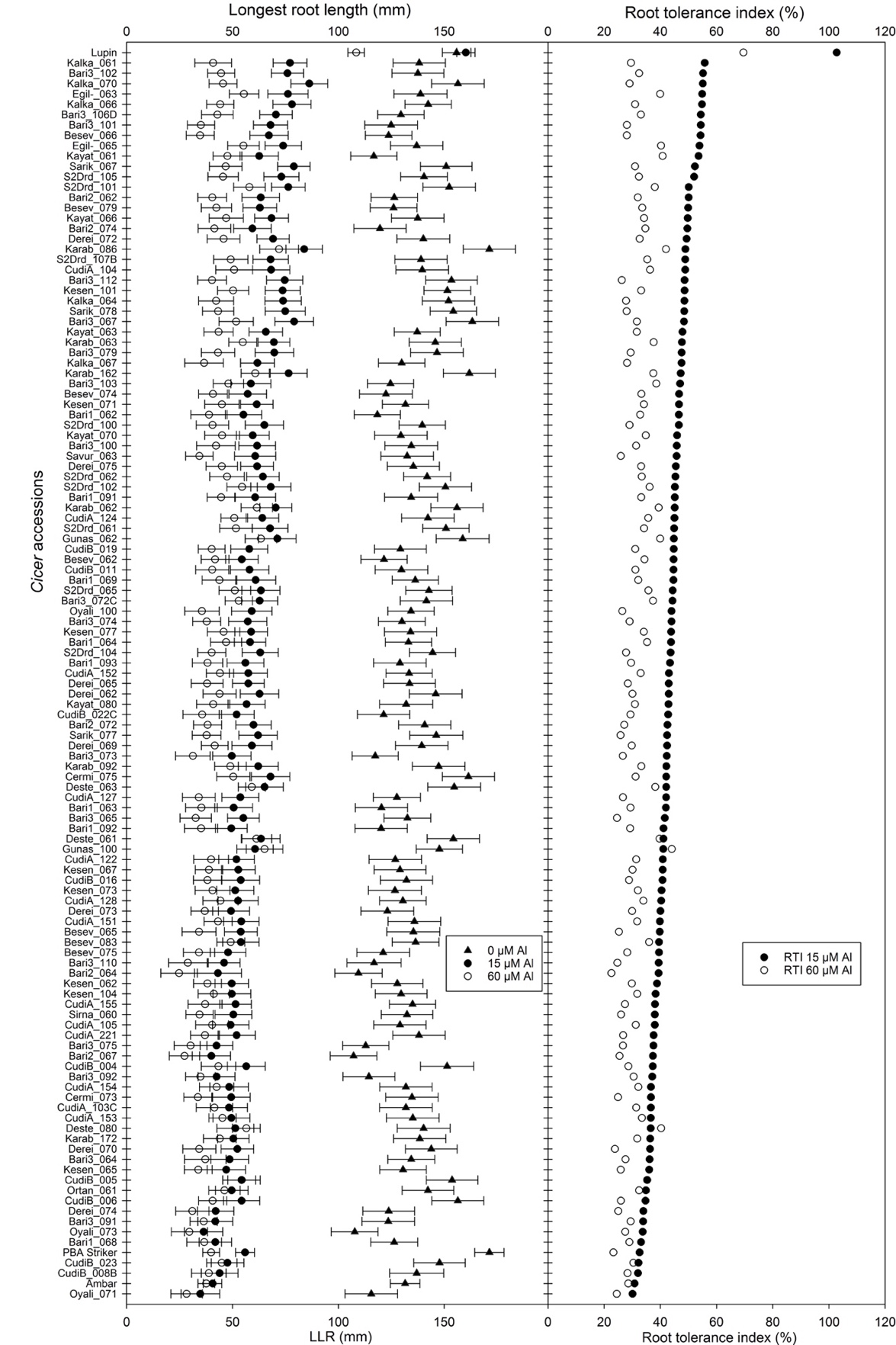


**Fig. D** Length of longest root (mm) and root tolerance index (RTI) (%) of 118 wild *Cicer* accessions, and Ambar, PBA Striker and lupin as check at 0, 15 and 60 μM Al screened in Experiment 5. See Table 1 for the species classification and more information on accessions screened.


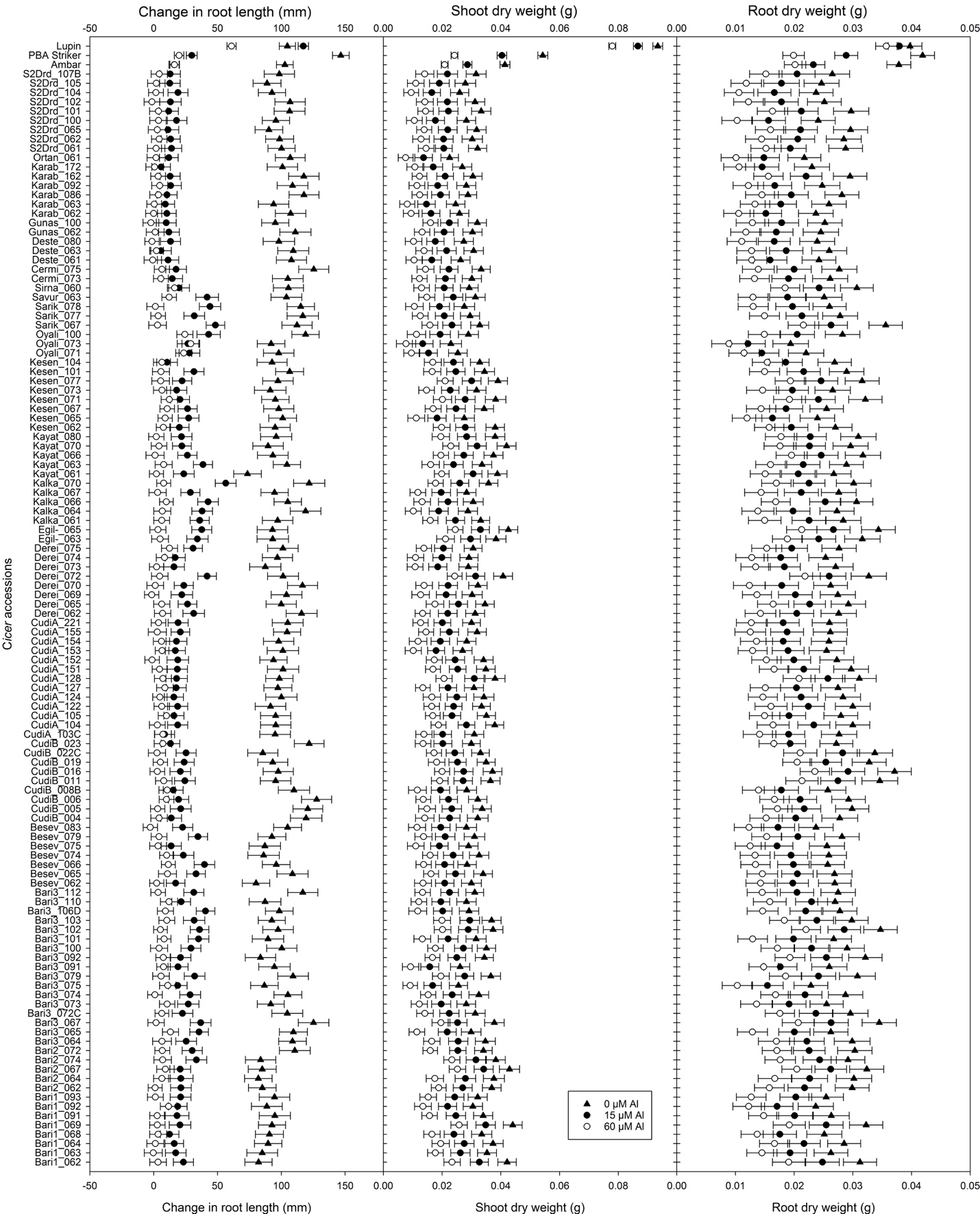


**Fig. E** The change in root length (mm), shoot and root dry weight (g) of 118 wild *Cicer* accessions, and Ambar, PBA Striker and lupin as check at 0, 15 and 60 μM Al screened in Experiment 5. See Table- 1 for the species classification and more information on accessions screened.


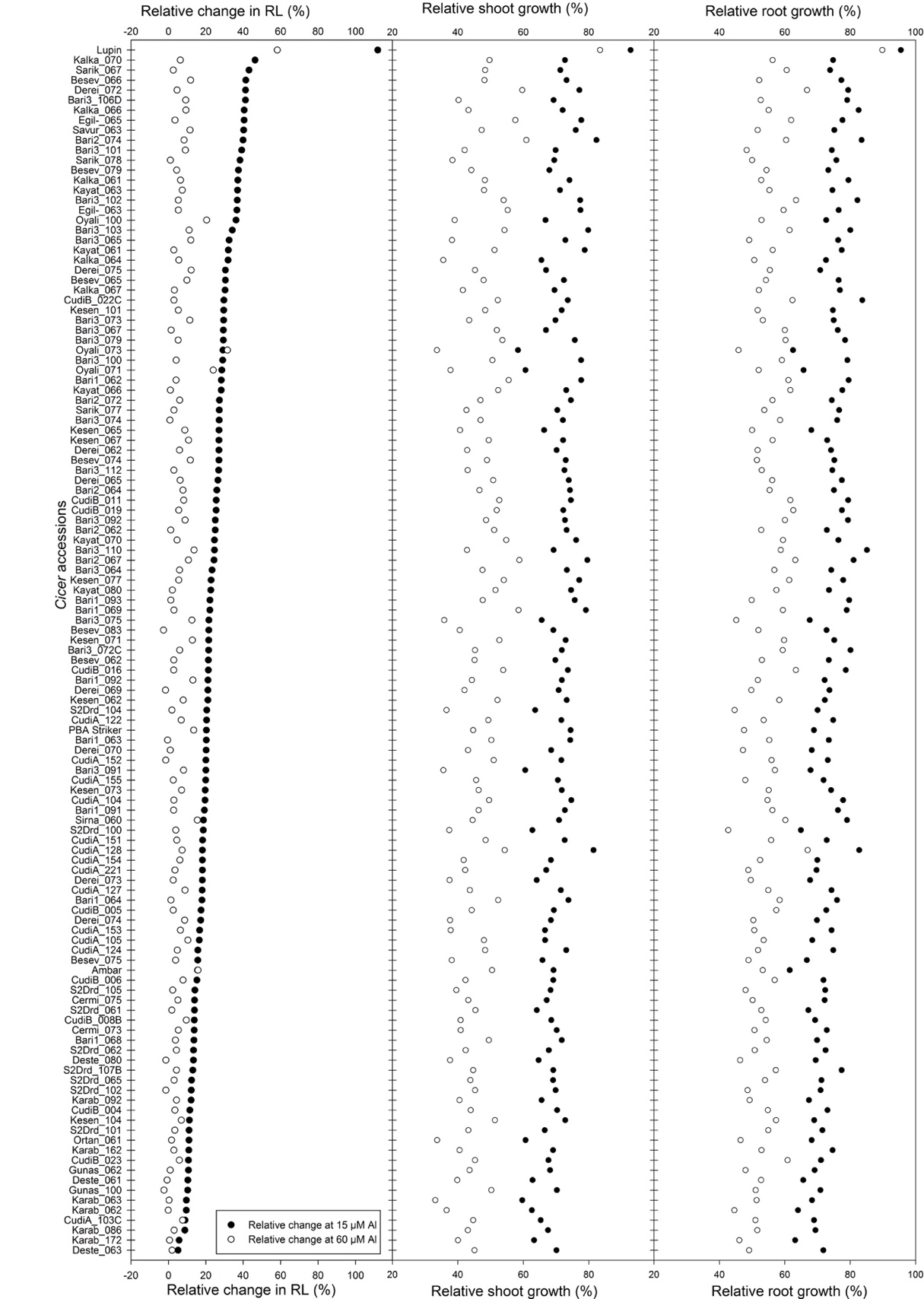


**Fig. F** Relative change in root length (%) (RRL), relative shoot growth (%) (RSG) and root growth (RRG) (%) of 118 wild *Cicer* accessions, and Ambar, PBA Striker and lupin as check at 0, 15 and 60 μM Al screened in Experiment 5. See Table 1 for the species classification and more information on accessions screened.


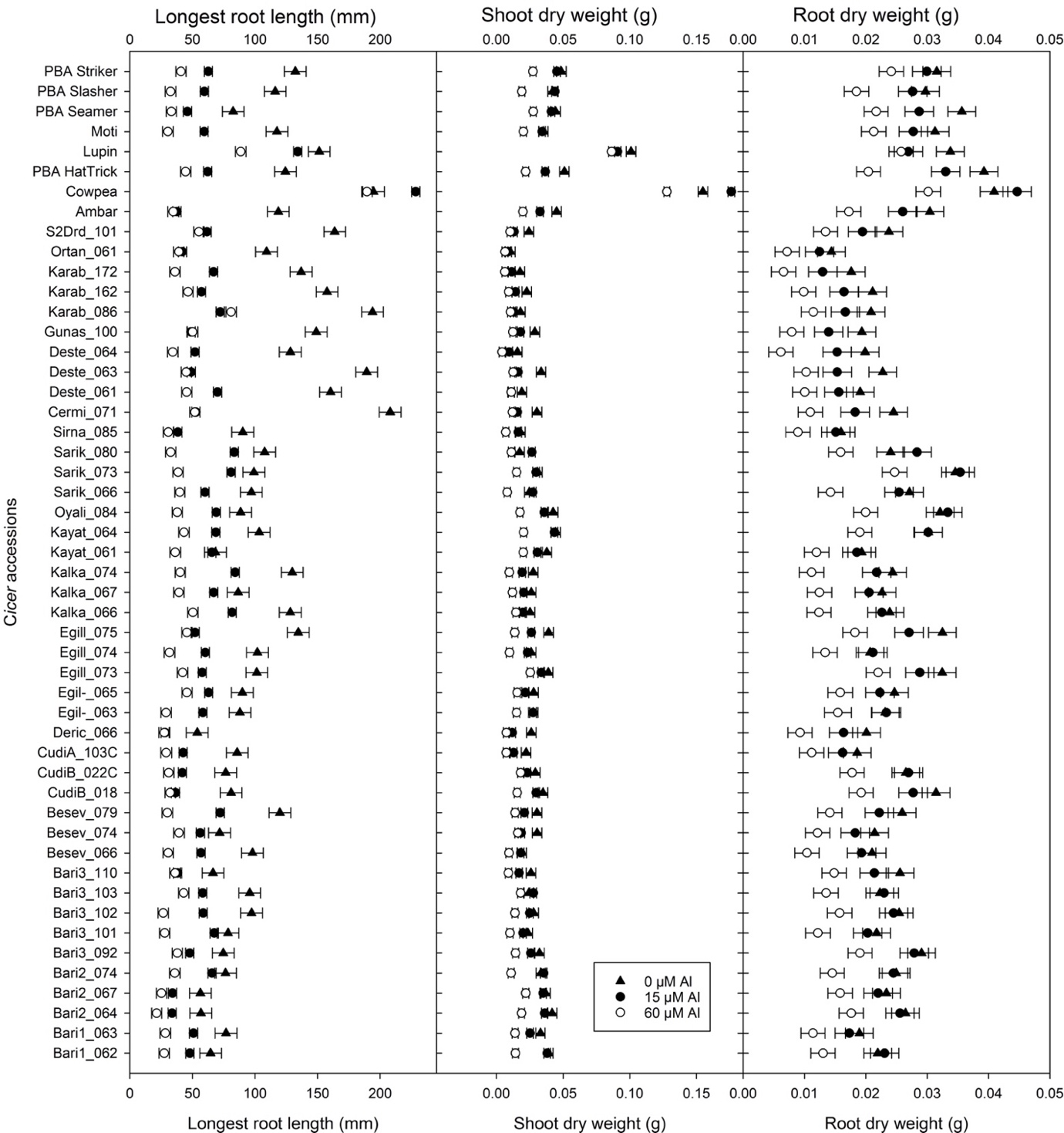


**Fig. G** The longest root length (mm), shoot and root dry weight (g) of 42 wild *Cicer* accessions, 6 domestic cultivars, lupin and cowpea as checks at 0, 15 and 60 μM Al screened in Experiment 6 (confirmation screening). See Table- 1 for the species classification and more information on accessions screened.
